# Targeted next generation sequencing identifies functionally deleterious germline mutations in novel genes in early-onset/familial prostate cancer

**DOI:** 10.1371/journal.pgen.1007355

**Published:** 2018-04-16

**Authors:** Paula Paulo, Sofia Maia, Carla Pinto, Pedro Pinto, Augusta Monteiro, Ana Peixoto, Manuel R. Teixeira

**Affiliations:** 1 Cancer Genetics Group, IPO Porto Research Center (CI-IPOP), Portuguese Oncology Institute of Porto (IPO Porto), Porto, Portugal; 2 Department of Genetics, Portuguese Oncology Institute of Porto (IPO Porto), Porto, Portugal; 3 Biomedical Sciences Institute Abel Salazar (ICBAS), University of Porto, Porto, Portugal; Cleveland Clinic Genomic Medicine Institute, UNITED STATES

## Abstract

Considering that mutations in known prostate cancer (PrCa) predisposition genes, including those responsible for hereditary breast/ovarian cancer and Lynch syndromes, explain less than 5% of early-onset/familial PrCa, we have sequenced 94 genes associated with cancer predisposition using next generation sequencing (NGS) in a series of 121 PrCa patients. We found monoallelic truncating/functionally deleterious mutations in seven genes, including *ATM* and *CHEK2*, which have previously been associated with PrCa predisposition, and five new candidate PrCa associated genes involved in cancer predisposing recessive disorders, namely *RAD51C*, *FANCD2*, *FANCI*, *CEP57* and *RECQL4*. Furthermore, using *in silico* pathogenicity prediction of missense variants among 18 genes associated with breast/ovarian cancer and/or Lynch syndrome, followed by KASP genotyping in 710 healthy controls, we identified “likely pathogenic” missense variants in *ATM*, *BRIP1*, *CHEK2* and *TP53*. In conclusion, this study has identified putative PrCa predisposing germline mutations in 14.9% of early-onset/familial PrCa patients. Further data will be necessary to confirm the genetic heterogeneity of inherited PrCa predisposition hinted in this study.

## Author summary

Prostate cancer (PrCa) is the most frequent cancer diagnosed in men worldwide, estimated to be responsible for the death of 27,540 men in the United States in 2015. Contrarily to other cancer types, the genetic contribution to the 10–20% of PrCa cases occurring in families with aggregation of the disease is largely unknown. Germline mutations in the *BRCA2* and the *MSH2* breast and colon cancer predisposing genes, respectively, explain only about 1.5% of our early-onset/familial PrCa cases. Taking advantage of recent deep sequencing technologies and an analysis pipeline established in our group, we have screened 121 PrCa patients with strong evidence of an hereditary component for mutations in 94 genes involved in several cancer predisposing syndromes. We found truncating/functionally deleterious mutations in seven genes and “likely pathogenic” missense variants in four genes, of which five and one, respectively, have not been previously associated with PrCa predisposition. We believe this study significantly contributes to the understanding of the genetic heterogeneity behind early-onset/familial PrCa.

## Introduction

Prostate cancer (PrCa) is the most frequent non-cutaneous cancer diagnosed in men worldwide and the third leading cause of male cancer deaths in Europe [1]. Despite efforts in early detection and screening strategies [2], PrCa is estimated to be responsible for the death of 27,540 men in the United States in 2015 [1]. Contrarily to other cancer types, very little is known about the genetic contribution to the 10–20% of PrCa cases with evidence of familial clustering [3]. In fact, besides age and race, family history is the only other well-established risk factor for PrCa [4]. While familial PrCa is defined by an aggregation of PrCa in families, hereditary prostate cancer (HPC) is characterized by a pattern of Mendelian inheritance associated with rare mutations in susceptibility genes [3,5]. First-degree relatives of a PrCa patient have a two-fold increased risk of developing the disease compared to the general population. The risk is even higher when the number of affected relatives increases and the age at diagnosis decreases [3,5,6]. The existence of a genetic component behind PrCa development is strengthened by the four-fold higher concordance rate of PrCa among monozygotic twins compared to dizygotic twins [7,8].

Linkage analysis and genome-wide association studies have pinpointed some *loci* associated with PrCa predisposition, but the majority has not been consistently reproduced [9]. In 2004, a combined genome-wide linkage analysis of 426 families from four HPC studies identified a *locus* at 17q21-22 strongly associated with PrCa [10]. Despite previous reports linking mutations in *BRCA1* (at 17q22) with PrCa predisposition [11,12], Ewing *et al*. later identified a rare but recurrent mutation (G84E) in the *HOXB13* gene (at 17q21) in up to 3% of the patients with both early-onset and family history of the disease, using a next-generation sequencing (NGS) approach covering the 202 genes present in the defined region of interest (ROI) at the 17q21-22 *locus* [13]. An increased risk of PrCa for the *HOXB13* G84E mutation carriers has been confirmed by several groups [14,15] and other *HOXB13* variants associated with PrCa have been found in other populations [16,17]. Besides *HOXB13*, *BRCA2* mutation carriers are also at increased risk of developing PrCa [18–20]. Overall, *BRCA2* mutations seem to explain about 2% of early-onset PrCa cases [19], a frequency that can be slightly higher for *BRCA2* mutations with a founder effect in specific populations [21,22]. Additionally, a higher risk for PrCa in Lynch syndrome families has been proposed [23,24], with some studies reporting a five- to ten-fold increased risk of PrCa development for carriers of *MSH2* mutations compared to non-carriers [25,26]. However, recent studies of our group found germline mutations in *HOXB13*, *BRCA2* and *MSH2* in only 1.5% of early-onset and/or familial PrCa cases [17,27].

Mutations in a few additional genes or specific variants, namely in *CHEK2* [28–31], *NBN* [32,33], *ATM* [34,35], and *BRIP1* [36], have been reported to increase the risk of PrCa, although some in a population-specific context. Despite these reports, the large majority of prostate carcinomas showing Mendelian inheritance still have no explanation concerning highly penetrant susceptibility variants. In this work, we aimed to evaluate the proportion of cases with early-onset and/or familial/hereditary PrCa that can be attributed to mutations in 94 genes associated with inherited cancer predisposition, using our validated targeted next generation sequencing (NGS) pipeline [37]. This approach allowed to identify functionally deleterious/“potentially pathogenic” mutations in nine genes, revealing six genes (*CEP57*, *FANCD2*, *FANCI*, *RAD51C*, *RECQL4* and *TP53*) not previously associated with PrCa predisposition. Overall, a candidate disease-causing mutation in a cancer predisposing gene was identified in 18 patients (14.9%), with *ATM* and *CHEK2* representing 61.1% of the cases.

## Results

### Truncating/Deleterious mutations in known PrCa risk genes

**Fig 1:**
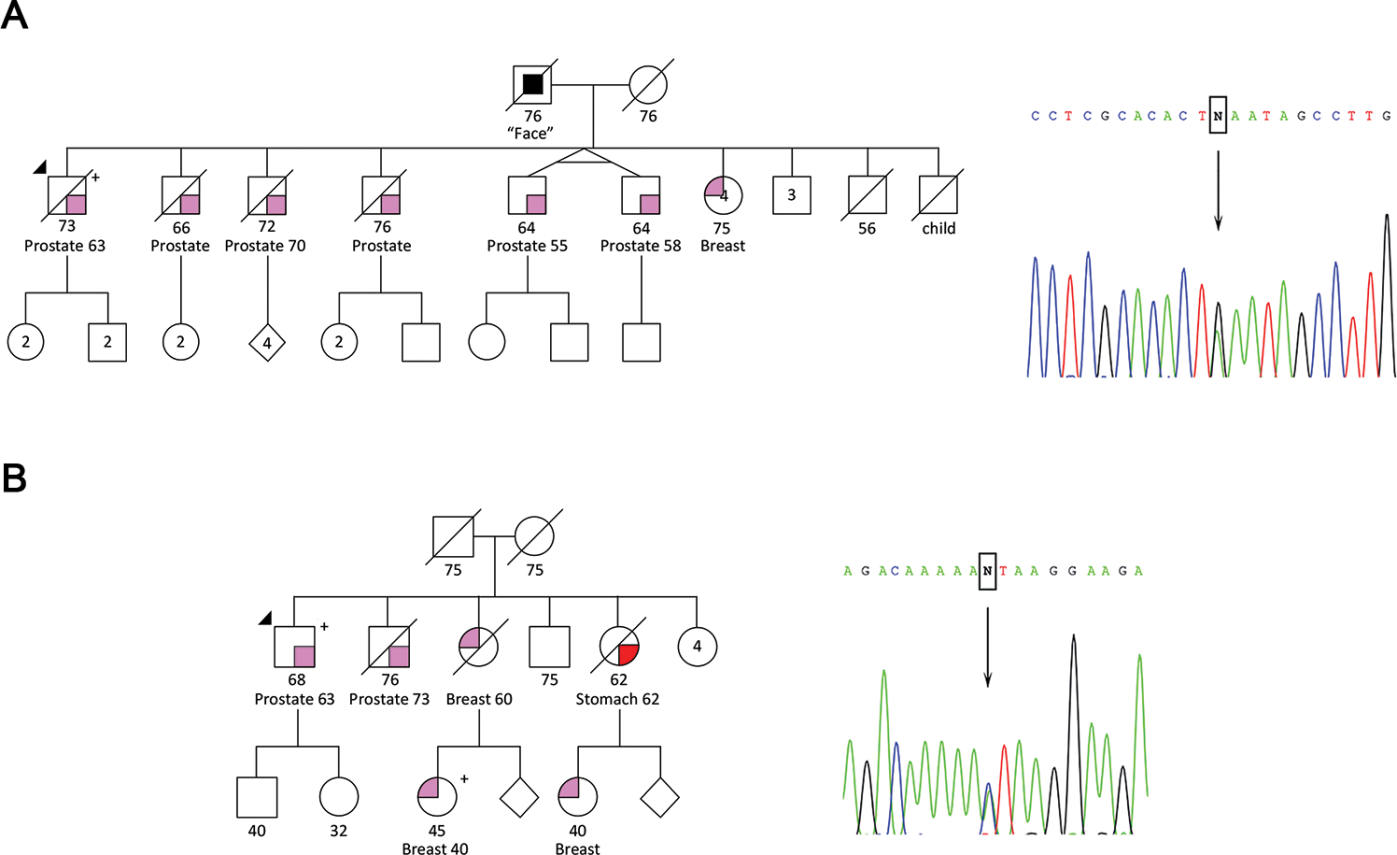
Pedigrees of patients carrying truncating/deleterious mutations in the known PrCa risk genes *ATM* and *CHEK2*. (A) Patient HPC177 harboring the *ATM* stop mutation c.652C>T. (B) Patient HPC395 harboring the *CHEK2* splicing mutation c.593-1G>T. Electropherograms of the Sanger sequencing validations are shown.

Of the genes previously reported to increase the risk for PrCa development (after excluding cases with known mutations in *HOXB13*, *BRCA2* and *MSH2* in this series), we found a nonsense mutation in *ATM* and a splicing mutation in *CHEK2* (Table 1). The *ATM* mutation c.652C>T, which leads to a premature stop codon at codon 218, was found in a patient (HPC177) with five brothers diagnosed with PrCa (Fig 1A), including twin brothers diagnosed before the age of 61 years, thus fulfilling the A1 and A2 criteria (see Material and Methods section for criteria description). The family is living abroad, which renders segregation analysis difficult to perform. The *CHEK2* mutation c.593-1G>T, predicted to affect the splice site by three of the four queried *in silico* predictors (S1 Table) and reported as “likely pathogenic” in ClinVar, was found in a patient (HPC395) with a family history of three breast cancer (BrCa) cases, two of them diagnosed at early age (Fig 1B), thus fulfilling the B3 criterion. One of the nices with BrCa is carrier of the *CHEK2* mutation c.593-1G>T.

**Table 1:** Truncating/Deleterious mutations found in the 121 cases by targeted NGS panel.

Gene	Variant position (GRCh37)	RefSeq Transcript^i^	cDNA change	Protein change	dbSNP ID	Sample	Fulfilled criteria
***Known PrCa risk genes***							
***ATM***	11:108114835	NM_000051.3	c.652C>T	p.(Gln218Ter)	N/A	HPC177	A1,A2
***CHEK2***	22:29115474	NM_007194.3	c.593-1G>T	p.(?)	rs786203229	HPC395	B3
***New PrCa risk genes***							
*Fanconi anemia genes*							
***FANCD2***	3:10109003	NM_001018115.1	c.2494+2T>C	p.(?)	rs779552164	HPC447	B3
***FANCI***	15:89803992	NM_001113378.1	c.206del	p.(Tyr69SerfsTer17)	N/A	HPC150	A2
***RAD51C***	17:56798156	NM_058216.2	c.890_899del	p.(Leu297HisfsTer2)	N/A	HPC186	B1,B2
*Genes of other recessive disorders*							
***CEP57***	11:95555126	NM_014679.4	c.791C>G	p.(Ser264Ter)	rs368470481	HPC421	B2
***RECQL4***	8:145738349	NM_004260.3	c.2636del	p.(Pro879LeufsTer69)	N/A	HPC455	B2

^i^ Available at www.ncbi.nlm.nih.gov/refseq/.

N/A- not available.

To strengthen the causality between these mutations and cancer development, we used KASP genotyping in 710 healthy controls and searched for the variant among 504 samples from non-prostate cancer cases analyzed with the same NGS panel and pipeline in the Department of Genetics of IPO Porto. Among the 504 cancer cases, the same *ATM* stop mutation was found in a patient diagnosed with bilateral BrCa at early age (previously described [38]) and the same *CHEK2* splicing mutation was found in an early-onset breast and colon cancer patient. No carriers were found either among our 710 healthy controls or in ExAC, for both mutations.

### Truncating/Functionally deleterious mutations in new candidate PrCa risk genes

#### Mutations in Fanconi anemia genes

Fanconi anemia (FA) is a recessive disorder caused by biallelic mutations in one of the (so far) nineteen FANC genes [39,40], of which only two (*RAD51* and *UBE2T*) are not covered by the TruSight Cancer panel. Along with *BRCA1* (*FANCS*) and *BRCA2* (*FANCD1*), involved in hereditary breast/ovarian cancer (HBOC) syndrome, three other members of the FANC family have been associated with an increased risk of development of BrCa and/or ovarian cancer (OvCa), namely *BRIP1* (*FANCJ*), *PALB2* (*FANCN*) and *RAD51C* (*FANCO*) [41–44]. We found a frameshift mutation in *RAD51C* and deleterious mutations, one splicing and one frameshift, in two FANC members not previously associated with cancer risk, *FANCD2* and *FANCI*, respectively (Table 1). In the ExAC database, the *FANCD2* splicing mutation is reported in a single case of Latin origin, whereas the other mutations were not found in ExAC or any of the other queried databases.

**Fig 2:**
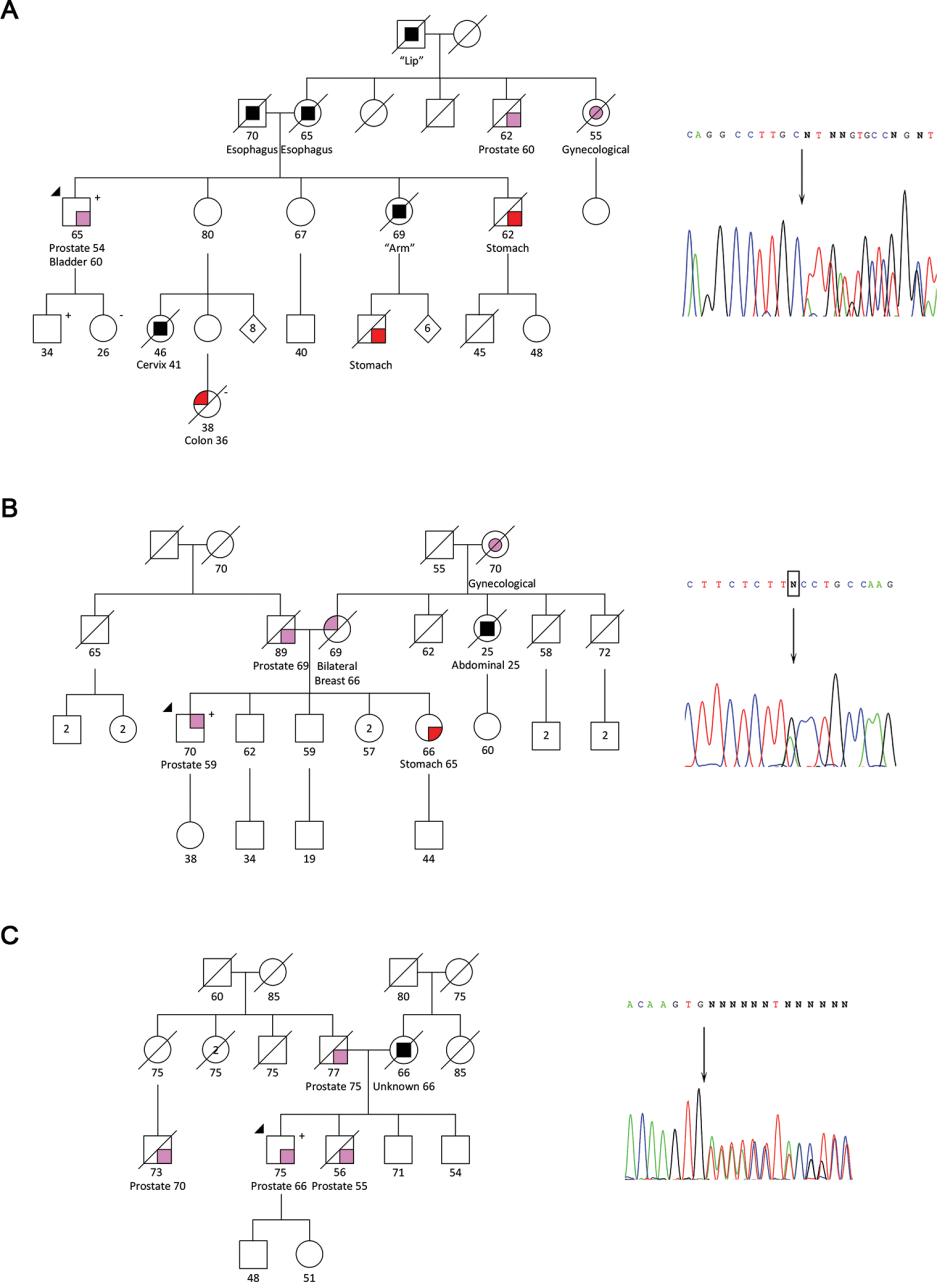
Pedigrees of patients carrying truncating/deleterious mutations in new candidate PrCa risk genes involved in Fanconi anemia. (A) Patient HPC186 harboring the *RAD51C* frameshift mutation c.890_899del. (B) Patient HPC447 harboring the *FANCD2* splicing mutation c.2494+2T>C. (C) Patient HPC150 harboring the *FANCI* frameshift mutation c.206del. Electropherograms of the Sanger sequencing validations are shown.

The *RAD51C* frameshift mutation c.890_899del is expected to result in a premature stop codon, and, consequently, in the loss of 80 amino acids at the protein C-terminal (predicted by MutationTaster), including the nuclear localization motif (Uniprot database). This mutation was found in a patient (HPC186) also diagnosed with bladder cancer (B2 criterion) and with a family history of early-onset cancer in several relatives (B1 criterion) (Fig 2A).

The *FANCD2* splicing mutation c.2494+2T>C is predicted to affect the splice site by three of the four queried *in silico* analysis tools (S1 Table). The patient harboring this mutation (HPC447) has a family history of five first- or second-degree relatives with cancer, fulfilling the B3 criterion for the bilateral BrCa in the mother and the early-onset abdominal cancer in the maternal aunt (Fig 2B).

The *FANCI* frameshift mutation c.206del is predicted to lead to a premature stop codon at codon 85 by MutationTaster, with the derived transcript probably being targeted to nonsense-mediated decay (NMD). The carrier patient (HPC150) has a family history of PrCa, fulfilling the A2 criterion (Fig 2C).

The screening for these mutations in the 710 healthy controls and in the 504 non-prostate cancer cases, as described above, showed absence of the mutations in the control samples and association of the *RAD51C* mutation c.890_899del with non-prostate cancer development in two patients and of the *FANCD2* mutation c.2494+2T>C in one. No other patients were found to carry the *FANCI* mutation c.206del.

#### Mutations in other genes involved in recessive disorders

**Fig 3:**
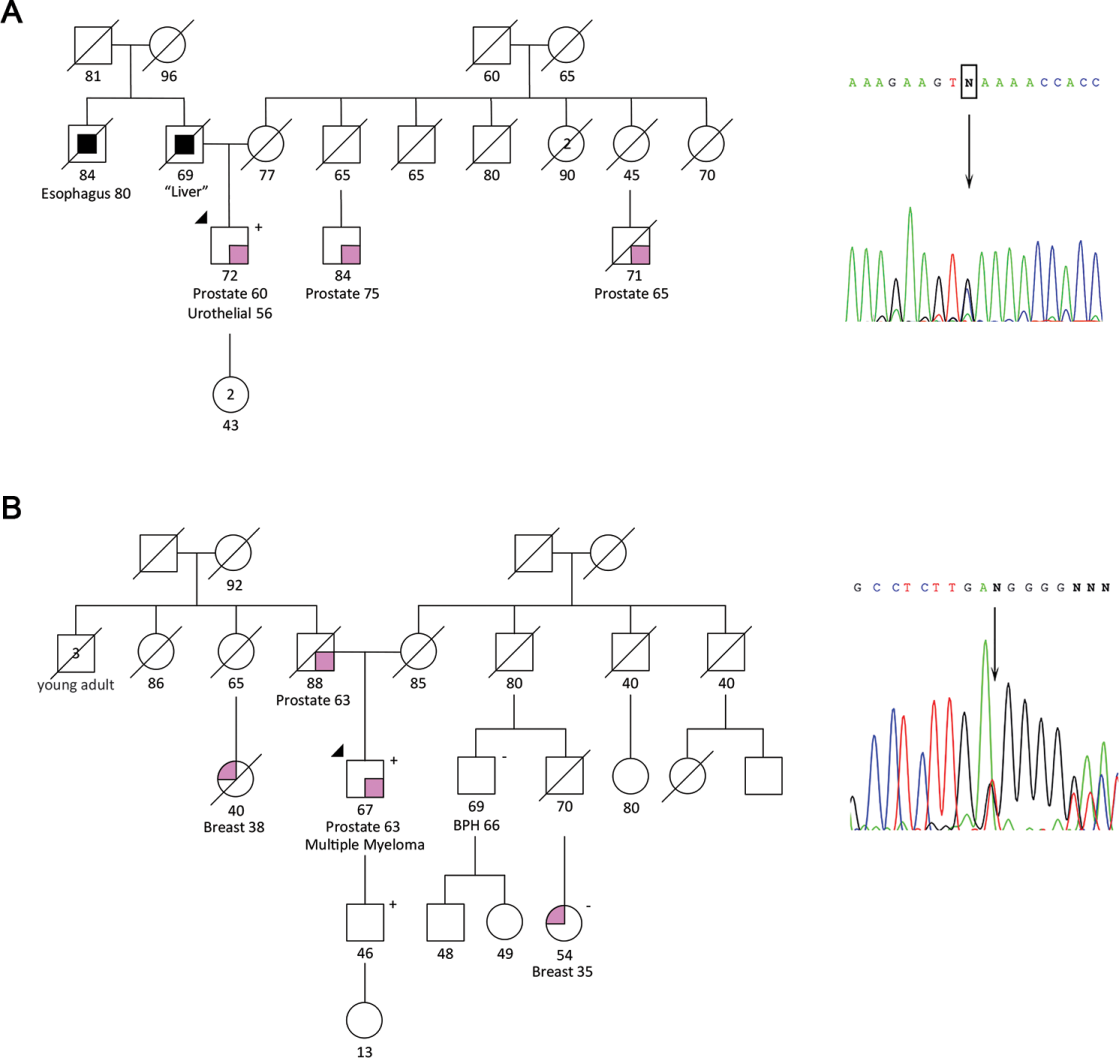
Pedigrees of patients harboring truncating/deleterious mutations in new candidate PrCa risk genes involved in other recessive disorders. (A) Patient HPC421 harboring the *CEP57* nonsense mutation c.791C>G. (B) Patient HPC455 harboring the *RECQL4* frameshift mutation c.2636del. Electropherograms of the Sanger sequencing validations are shown.

We found a nonsense mutation (c.791C>G) in *CEP57*, a gene involved in the mosaic variegated aneuploidy syndrome 2 (MVA2; OMIM #614114), in patient HPC421. This mutation is expected to encode a C-terminal truncated protein at the residue 264. According to the Uniprot database, this codon is located upstream of the known functional domains of the centrosomal protein of 57kDa encoded by this gene, which are expected to be lost with the mutation if the mRNA escapes NMD, as predicted by MutationTaster. The patient harboring this mutation was diagnosed with PrCa four years after the diagnosis of an urothelial cancer, thus fulfilling the B2 criterion (Fig 3A).

Additionally, we found a frameshift mutation in *RECQL4*, a gene involved in three recessive syndromes with overlapping features, the Rothmund-Thomson syndrome (RTS; OMIM #268400), the Baller-Gerold syndrome (BGS; OMIM #218600), and the RAPADILINO syndrome (OMIM #266280). The *RECQL4* frameshift mutation c.2636del was found in patient HPC455, who has also multiple myeloma (B2 criterion) (Fig 3B). MutationTaster is not able to compute predictions for *RECQL4* mutations due to the lack of a reliable protein-coding transcript. In the Uniprot database, the described functional domains of the ATP-dependent helicase encoded by this gene are upstream of the c.2636del mutation.

The *CEP57* nonsense mutation c.791C>G is only described in one case in the Exome Variant Server (rs368470481), whereas the *RECQL4* frameshift mutation c.2636del is not described in any of the queried databases. None of these mutations was found either among our 710 healthy controls or among the 504 non-prostate cancer cases screened as described above.

### “Likely/potentially pathogenic” variants in genes predisposing to breast/ovarian cancer and/or Lynch syndrome

Considering that most of the genes so far associated with an increased risk for PrCa development have previously been described to predispose to breast/ovarian cancer and/or Lynch syndrome, we looked for missense variants in the 18 genes associated with these diseases, namely *ATM*, *BLM*, *BRCA1*, *BRCA2*, *BRIP1*, *CDH1*, *CHEK2*, *MLH1*, *MSH2*, *MSH6*, *NBN*, *PALB2*, *PMS2*, *PTEN*, *RAD51C*, *RAD51D*, *STK11*, and *TP53*. Missense variants predicted to be pathogenic by at least 12 of the 15 *in silico* pathogenicity predictors (including at least three conservation tools) were considered “potentially pathogenic”. Of the 42 missense variants found (S2 Table [45]), ten variants fulfill these criteria (Table 2) and include four variants in *ATM*, two in *CHEK2*, and one in each of the genes *BRIP1*, *MSH2*, *MSH6* and *TP53*. The *CHEK2* missense mutation c.349A>G, found in two patients (HPC188 and HPC289), was the only mutation classified as “pathogenic/likely pathogenic” in ClinVar. Patient HPC188 has family history of PrCa, with three first- and second-degree relatives diagnosed at or before the age of 65 years (one early-onset), thus fulfilling the A1 and A2 criteria (S1A Fig). Patient HPC289 is an early-onset PrCa case, fulfilling the B1 and B3 criteria for having a heavy family history of cancer, with several cases diagnosed at early age (S1B Fig). Both *MSH2* and *MSH6* variants are reported in public databases and classified as variants of unknown significance (VUS) in ClinVar. As mutations in Lynch syndrome predisposing genes are usually associated with loss of protein expression in the tumor, we performed immunohistochemistry for the MSH2 and MSH6 proteins in the prostate tumors of the patients HPC371 and HPC332, respectively, and no loss of expression was found, rendering the *MSH2* and *MSH6* mutations as probably not associated with PrCa development in these patients. Apart from the *CHEK2* missense mutation c.349A>G, the remaining missense variants here identified were either not described in the literature or classified as VUS. To increase our understanding on the pathogenic potential of these variants, we screened our 710 healthy controls and the 504 non-prostate cancer samples as described above. For the *ATM* mutations c.995A>G and c.8560C>T and for the *TP53* mutation c.839G>A, no additional carriers were found. The *BRIP1* mutation c.847T>C and the *CHEK2* mutation c.695G>T were found in one of the 504 cancer cases (each) and the *ATM* mutations c.1595G>A and c.5750G>A were found in two cases (each) of the 710 healthy controls and in six and three cases, respectively, of the 504 cancer cases. Of all these variants, only the *ATM* mutation c.8560C>T was found significantly increased in our PrCa patients comparing with our healthy controls (*P* = 0.024; S3 Table), with the *CHEK2* mutation c.349A>G reaching borderline significance (*P* = 0.057). With the exception of the *TP53* mutation c.839G>A, not found in any of the 504 non-prostate cancer patients, all missense mutations have no significant frequency differences in cancer patients fulfilling criteria for other hereditary cancer syndromes (*P*>0.05; S3 Table). When comparing the frequencies obtained in our PrCa patients with those of the Non-Finnish Europeans (NFE) described in ExAC, highly significant associations are obtained for all the *ATM* and *CHEK2* missense mutations (S3 Table). Following the guidelines from the American College of Medical Genetics and Genomics and the Association for Molecular Pathology (ACMG-AMP) for variant interpretation and classification [46] using InterVar [47], all the missense variants here identified in *ATM*, *CHEK2*, *BRIP1* and *TP53* are classified as VUS. Adding the PS3 criterion (“well-established *in vitro* or *in vivo* functional studies supportive of a damaging effect on the gene or gene product”) to the classification of the *CHEK2* variant c.349A>G [48,49] and the PS4 criterion (“the prevalence of the variant in affected individuals is significantly increased compared with the prevalence in controls”) to the classification of the *ATM* variant c.8560C>T, supports the “likely pathogenic” nature of these variants in PrCa development.

**Table 2:** “Likely/Potentially pathogenic” missense variants found in the 121 cases by targeted NGS panel.

Gene	Variant position (GRCh37)	RefSeq Transcript^i^	cDNA change	Protein change	dbSNP ID	Sample	Fulfilled criteria	Fulfilled ACMG-AMP criteria^ii^
***ATM***	11:108117784	NM_000051.3	c.995A>G	p.(Tyr332Cys)	N/A	HPC238	A1,A2,A3	PM2, BP1
***ATM***	11:108121787	NM_000051.3	c.1595G>A	p.(Cys532Tyr)	rs35963548	HPC167	B2	PM2, PP3, BP1
						HPC400	B1	
***ATM***	11:108178699	NM_000051.3	c.5750G>A	p.(Arg1917Lys)	N/A	HPC20	B1	PM2, PP3, BP1
***ATM***	11:108216611	NM_000051.3	c.8560C>T	p.(Arg2854Cys)	rs201958469	HPC3	A3	**PS4**, PM1, PM2, PP3, BP1
						HPC186	B1,B2	
						HPC332	A3,B1	
***BRIP1***	17:59885899	NM_032043.2	c.847T>C	p.(Cys283Arg)	N/A	HPC118	A2	PM2, PP3, BP1
***CHEK2***	22:29121326	NM_007194.3	c.349A>G	p.(Arg117Gly)	rs28909982	HPC188	A1,A2	**PS3**, PM2, PP3, PP5
						HPC289	B1,B3	
***CHEK2***	22:29107994	NM_007194.3	c.695G>T	p.(Gly232Val)	rs779322187	HPC89	B2	PM1, PM2, PP3
MSH2*	2:47693857	NM_000251.2	c.1571G>A	p.(Arg524His)	rs63751207	HPC371	B3	N/D
MSH6*	2:48026851	NM_000179.2	c.1729C>T	p.(Arg577Cys)	rs542838372	HPC332	A3,B1	N/D
***TP53***	17:7577099	NM_000546.5	c.839G>A	p.(Arg280Lys)	N/A	HPC394	A1,A2,B2	PM2, PP3

*no loss of expression was observed in the PrCa tissue by immunohistochemistry.

N/A- not available; N/D- not determined.

^i^ Available at www.ncbi.nlm.nih.gov/refseq/;

^ii^ According to InterVar (http://wintervar.wglab.org/); manually introduced criteria are highlighted in bold letters.

### Clinicopathological associations of mutation carriers

Of the 121 cases enrolled in this study, 45 have criteria to be classified as familial/hereditary PrCa (A group) and 86 are cases of early-onset PrCa and/or PrCa associated with clustering of other cancers in the family (group B), with ten cases fulfilling both A and B criteria (S4 Table). Regarding age at diagnosis, 64 cases (52.9%) were diagnosed with PrCa at or before the age of 55 years, thus being considered early-onset PrCa cases. Considering the number of cases with prostate carcinomas in the 121 families, 91 cases (75.2%) have family history of two or more relatives with PrCa, with 27 cases (22.3%) having three and 33 cases (27.3%) having at least four.

When comparing clinicopathological characteristics of the patients harboring the deleterious/”potentially pathogenic” mutations (n = 18; excluding the cases with the *MSH2* and *MSH6* VUS, described above) with the “negative” group (n = 103), no statistically significant associations were observed, either considering all cases or considering the subgroups of cases with familial/hereditary PrCa or early-onset PrCa (S5 Table).

### Incidental findings

Consistent with the increasing chance of incidental findings of the NGS approaches, we found a c.3846_3860del in-frame deletion in *MSH6* that falls into this classification. This mutation was found in the patient carrying also the truncating mutation in *RAD51C* (HPC186) and is classified as pathogenic in two of the Lynch syndrome families diagnosed at IPO Porto. However, we find unlikely its association with the PrCa in this patient, as no loss of MSH6 expression was observed in the tumor (contrarily to what we observed in the colon carcinomas of our Lynch syndrome families). Analyses in the available relatives showed segregation of the variant in the niece with colon cancer (S2 Fig).

## Discussion

In this work we used a NGS approach targeting the full coding-sequence of 94 genes associated with cancer predisposition to identify germline mutations in a selected series of 121 PrCa patients with early-onset disease and/or criteria for familial/hereditary PrCa, alone or associated with other cancers. This strategy is justifiable by the fact that, with the exception of *HOXB13*, all the genes so far associated with PrCa hereditary predisposition were previously associated with an increased risk for BrCa, OvCa or other cancers, including those causing the phenotypically heterogeneous diseases hereditary breast/ovarian cancer (HBOC) and Lynch syndrome [20,24,27,50]. Using our previously established NGS analysis pipeline [37], and after excluding the few cases in our series with germline mutations in *HOXB13* or in genes associated with HBOC or Lynch syndrome [17,27], we found monoallelic truncating/deleterious mutations in seven genes, of which only *ATM* and *CHEK2* have been previously implicated in PrCa development [28–31,34,35,43]. Deleterious mutations in *ATM* and *CHEK2* thus represent 0.8% (each) of the cases enrolled in this study, with the nonsense *ATM* mutation representing 2.2% of the cases fulfilling criteria for familial/hereditary PrCa (A group), which resembles the frequency of mutations found in *BRCA2* in earlier studies [27,35]. Curiously, both mutations occur in families with several BrCa cases and were both found in one case (each) of the 504 non-prostate cancer cases analyzed with the same NGS approach in the Department of Genetics for fulfilling criteria for HBOC.

We found monoallelic functionally deleterious mutations in three genes of the FA family, namely *RAD51C* (*FANCO*), *FANCD2* and *FANCI* genes, the latter two not previously associated with cancer risk. *RAD51C*, a *RAD51* paralog involved in the homologous recombination (HR) repair pathway [51], was first described as a susceptibility gene for BrCa and OvCa, showing complete segregation in six families [44]. Nowadays, *RAD51C* deleterious mutations are established as a risk factor for OvCa only, with a prevalence of about 0.8% in familial OvCa and 0.4–1.1% in OvCa cases unselected for family history [42]. The family history of the patient harboring the c.890_899del mutation in *RAD51C* has no confirmed ovarian cancer diagnosis, but includes a relative with gynecological cancer deceased at young age. FANCD2 and FANCI are involved in the initial steps of the FA pathway, leading to the activation of downstream repair factors, such as FANCD1 (BRCA2), FANCJ (BRIP1), FANCN (PALB2) and FANCO (RAD51C), to mediate HR [52]. Considering that mutations in all these four FA members have been associated with risk for BrCa and/or OvCa [41,43,44], with mutations in *BRIP1* and *BRCA2* also associated with PrCa development [19,20,36], our report of mutations in *RAD51C*, *FANCD2* and *FANCI* may increase to five the list of FA members involved in PrCa predisposition. In our series, functionally deleterious mutations in FA genes represent 4.4% (2/45) of the familial/hereditary PrCa cases (A criteria) and 1.6% (1/64) of the early-onset PrCa cases. Among the 504 non-prostate cancer cases diagnosed at our institution with the same NGS approach, three carriers of the same mutations were found, one with the *FANCD2* mutation and two with the *RAD51C* mutation, with different cancers occurring in the families. Further studies are required to determine the frequency of germline mutations in these genes in PrCa and other hormone-related cancers, as the pedigrees of both case HPC186 and case HPC447, with the *RAD51C* and the *FANCD2* functionally deleterious mutations, respectively, include relatives affected with BrCa and/or gynecological cancers.

To our knowledge, this is also the first report of heterozygous germline truncating mutations in *CEP57* and *RECQL4* as possible cancer risk factors. *CEP57* encodes a 57 kDa member of the CEP family of centrosomal proteins involved in MVA2, a rare pediatric syndrome with high risk of development of childhood cancers [53,54]. On the other hand, *RECQL4* belongs to a family of five RecQ helicases [*RECQL1*, *WRN* (*RECQL2*), *BLM* (*RECQL3*), *RECQL4* and *RECQL5*] [55]. Interestingly, monoallelic mutations in *RECQL1* and in the Bloom syndrome gene *BLM* have been described as risk factors for BrCa [56,57], although the *BLM* association has been contested by others [58,59]. According to the Uniprot database, the *RECQL4* frameshift mutation c.2636del we here describe is not expected to affect the known functional domains of the protein, but more downstream (C-terminal) mutations in *RECQL4* have been shown to cause RTS or GBS [55] and the C-terminal seems to be necessary for RECQL4 nucleolar localization through interaction with PARP-1 [60], therefore making very likely its deleterious nature. Additionally, the absence of both mutations in public databases, namely ExAC, and in the 504 non-prostate cancer cases analyzed in our institution with the same NGS approach, may reflect their PrCa specificity.

In addition to the seven cases with truncating/deleterious mutations, we found “likely/potentially pathogenic” missense mutations in 11 PrCa families. Taking into account the diversity and general high concordance of the *in silico* tools that were considered for the prediction of variant pathogenicity (S2 Table), along with the low frequency found among the 710 healthy control cases screened (S3 Table) and with the fact that other missense mutations in *ATM*, *CHEK2* and *TP53* have been linked with cancer development [61,62], it is plausible that the variants in *ATM*, *BRIP1*, *CHEK2* and *TP53* here identified may explain PrCa susceptibility in the families carrying them. The pathogenic nature of the *CHEK2* mutation c.349A>G found in two cases, was suggested in several studies, showing loss of DNA damage response and impaired activation due to lack of phosphorylation [48,49]. Furthermore, this *CHEK2* variant has been found in three of 694 *BRCA1*/*BRCA2*-negative BrCa families, two from the United Kingdom and one from the Netherlands, being described as a moderate to low penetrance variant [63]. On the other hand, in a large case-control study gathering data from three consortia participating in the Collaborative Oncological Gene-environment Study (COGS), the *CHEK2* variant c.349A>G was associated with increased BrCa risk (odds-ratio 2.26), but not with an increased risk for PrCa or OvCa [43]. Segregation analysis and/or phenotypic evaluation *in vitro* would be useful to complement the available information concerning the pathogenicity of this and the remaining missense variants here identified. For the variants found significantly associated (or showing borderline significance) with PrCa development, namely the *ATM* variant c.8560C>T and the *CHEK2* variant c.349A>G, larger cohorts of familial/early-onset PrCa cases would be useful to define cancer risk estimates and the age-standardized PrCa risk attributed to these variants.

Looking at the overlap between the patients harboring truncating/functionally deleterious mutations and those harboring “likely/potentially pathogenic” missense variants, the *ATM* variant c.8560C>T, found in three patients (HPC3, HPC186 and HPC332), is the only variant overlapping with other mutations, namely in patient HPC186, who carries the *RAD51C* frameshift mutation, and in patient HPC332, who carries the *MSH6* c.1729C>T variant. Immunohistochemistry analysis for MSH6 in the tumor of patient HPC332 showed normal MSH6 expression, thus reducing the likelihood that the *MSH6* c.1729C>T variant is a PrCa risk factor and rendering the *ATM* mutation c.8560C>T the most likely risk variant in this patient. Excluding the case HPC186 (with co-occurrence of the *RAD51C* frameshift mutation), *ATM* represents the most commonly mutated gene in our series, eventually explaining increased risk of PrCa in seven cases (~5.8%), with *CHEK2* being the second most frequently mutated gene (four cases, ~3.3%).

Overall, functionally deleterious/“likely/potentially pathogenic” variants were found in 18 patients (excluding the two families with missense mutations in *MSH2* and *MSH6*). Of these, eight patients (44.4%) fulfill the A criteria and 12 (66.7%) fulfill the B criteria (two cases complying with both), representing 17.8% and 13.9% of the samples enrolled in each group. Seven of the 18 cases (38.9%) were diagnosed at early age, representing 10.9% of the patients in the early-onset group. Comparing clinicopathological data from patients harboring these variants with the group of patients without an identified “potentially pathogenic” mutation, no statistically significant associations were found.

In the context of this study, we identified one truncating variant in a gene that is included in the list of incidental findings recommended for return to patients after clinical sequencing by the guidelines of the American College of Medical Genetics and Genomics [64]. The previously unreported *MSH6* in-frame mutation c.3846_3860del predisposes to Lynch syndrome (OMIM #120435), as it has been classified as pathogenic in two Lynch syndrome families in our institution, with evidence that included demonstration of loss of expression restricted to MSH6 in the colorectal tumors of carriers, a pattern also observed in the colon cancer of a relative of this patient who is also carrier of this in-frame *MSH6* variant. On the other hand, as no loss of MSH6 expression was observed in the prostate tumor, this *MSH6* variant is unlikely to explain the PrCa predisposition in this family, which is most likely related to the *RAD51C* truncation mutation or the *ATM* missense mutation also found in this patient. Even though targeted sequencing was performed under a research protocol and not as part of clinical sequencing, this incidental finding was reported to the patient during genetic counseling, as recommended by the guidelines of the American College of Medical Genetics and Genomics, and appropriate follow-up is being offered to the family as judged clinically appropriate.

In conclusion, we found functionally deleterious/“likely/potentially pathogenic” germline mutations in 18 of the 121 (14.9%) familial/hereditary and/or early-onset PrCa cases selected for this study. To our knowledge, this study is the first to report functionally deleterious germline mutations in the three FA genes *RAD51C*, *FANCD2* and *FANCI*, and in two genes until now only associated with recessive disorders, *CEP57* and *RECQL4*. Further data will be necessary to confirm the genetic heterogeneity of inherited PrCa predisposition hinted in this study.

## Material and methods

### Ethics statement

This study is in accordance with the ethical standards of the Ethics Committee of the Portuguese Oncology Institute of Porto (approval number 38.010) and with the 1964 Helsinki declaration and its later amendments or comparable ethical standards.

### Patient samples

We selected 121 cases from our previously described series of 462 early-onset and/or familial/hereditary PrCa cases [17], with two groups being considered: A) cases with familial/hereditary PrCa, and B) cases with early-onset PrCa and/or association with other types of cancer. Among the cases in the A group, three criteria were defined: 1) cases with at least three first-degree relatives with PrCa independently of the ages at diagnosis, 2) cases with two first-degree relatives with PrCa with average age at diagnosis ≤65 years and at least one of the affected cases diagnosed before the age of 61, and 3) cases diagnosed before the age of 61 with at least two first- or second-degree relatives with PrCa and average age at diagnosis of the three younger cases ≤65. Regarding the cases in the B group, three criteria were considered: 1) cases diagnosed before the age of 56 years with at least three first- or second-degree relatives diagnosed with cancer and average age at diagnosis of the three younger diagnoses ≤55, 2) cases diagnosed with second primary cancers besides PrCa and 3) cases with relatives diagnosed with either early-onset and/or rare cancer types (bilateral breast, male breast, brain) and/or clustering of other cancer types (e.g. breast, colon, or gastric cancers). Cases previously identified as harboring pathogenic mutations in known PrCa predisposing genes (*HOXB13*, *BRCA2* and *MSH2*) were excluded from this case priorization [17,27].

DNA previously extracted from peripheral blood leucocytes by standard procedures [17] was quantified using Qubit Fluorometer (Thermo Fisher Scientific, Waltham, MA, USA).

### Control samples

We used as control samples 710 healthy individuals (391 males and 319 females; mean age 55.1 years; SD±9.4 years), including 528 blood donors (285 males and 243 females) from the Portuguese Oncology Institute of Porto with no personal history of cancer at the time of blood collection and 182 healthy relatives (106 males and 76 females) with negative predictive genetic testing (each from independent families).

### Next generation sequencing

We applied our previously established NGS pipeline [37] using the TruSight Rapid Capture target enrichment workflow and the TruSight Cancer panel, both from Illumina, Inc. (San Diego, CA, USA). For variant analysis, sequences were aligned to the reference genome (GRCh37/hg19) using three different alignment and variant calling software: Isaac Enrichment (v2.1.0), BWA Enrichment (v2.1.0) and NextGENe (v2.4.1; Softgenetics, State College, PA, USA), as previously described [37]. Briefly, for variant annotation and filtering, .*vcf* (variant call format) files from the three software were imported into GeneticistAssistant (Softgenetics) and filtered for variant frequency in our *in-house* database, excluding variants present in more than 10% of the cases. Additional variant selection included those with coverage >20x, alternative variant frequency between 30% and 70% (excluding variants in mosaicism), and minor allele frequency (MAF) ≤0.1% [65,66]. Synonymous variants and intronic variants at more than 12-bp away from exon-intron boundaries were excluded. For MAF filtering, data was obtained from the 1000 Genomes Project [Based on Project Phase III Data [67]], Exome Variant Server [from NHLBI Exome Sequencing Project (http://evs.gs.washington.edu/EVS/), accessed in January, 2017] and Exome Aggregation Consortium [ExAC (http://exac.broadinstitute.org), accessed in January, 2017] databases, whenever available. Variants assigned as not pathogenic, likely not pathogenic, of no clinical significance or of little clinical significance, according to public databases, namely ClinVar (http://www.ncbi.nlm.nih.gov/clinvar/, accessed in January, 2017), Breast Cancer Information Core [BIC (https://research.nhgri.nih.gov/bic/), accessed in January, 2017)], and InSiGHT (via the Leiden Open-source Variation Database [LOVD (http://www.lovd.nl/3.0/home), accessed in January, 2017] [68]), were discarded.

### Sanger sequencing validation

All the variants identified were validated by Sanger sequencing. For this purpose, primers (S6 Table) were designed using the Primer-BLAST design tool from the National Center for Biotechnology Information (NCBI) [69]. For PCR amplification, an initial denaturation step was performed at 95°C for 15min, followed by 35 cycles with denaturation at 95°C for 30s, annealing at appropriate temperature (58–62°C) for 30s and extension at 72°C for 45s. A final extension step at 72°C for 9min was included. For the sequencing reaction, the BigDye Terminator v3.1 Cycle Sequencing Kit (Thermo Fisher Scientific) was used, according to the manufacturer’s instructions, and samples were run in a 3500 Genetic Analyzer (Thermo Fisher Scientific). For validation of the *RECQL4* variant, primers and PCR conditions from Nishijo *et al*. were used [70]. The *TP53* variant was validated following the IARC (International Agency for Research on Cancer) protocol for direct sequencing (http://p53.iarc.fr/, update 2010). Primers and PCR conditions for Sanger sequencing validation of *MSH2* and *MSH6* variants were kindly provided by Professor Michael Griffiths from the West Midlands Regional Genetics Laboratory, Birmingham Women’s NHS Foundation Trust, Birmingham, United Kingdom.

### *In silico* prediction of variant pathogenicity

To explore the functional consequence of truncating/deleterious variants, MutationTaster [71] and Uniprot [72] were queried. To infer the putative impact on splicing, the splice site predictors Human Splicing Finder 3.0 [73], MaxEntScan [74], NNSPLICE [75] and NetGene2 [76] were used. To predict the biological impact of missense mutations, we looked at data from the predictor tools embedded in the NGS Interpretative Workbench from GeneticistAssistant, which includes the functional predictors SIFT, PolyPhen2, LRT, MutationTaster, PROVEAN, FATHMM, CADD, MutationAssessor, MetaLR, MetaSVM and VEST3, and the conservation analysis tools PhyloP, GERP++, PhastCons and SiPhy, as previously described [37].

### Clinicopathological associations

To search for clinicopathological associations between mutation carriers and non-carriers, information on PSA at diagnosis, tumor staging and Gleason Score were gathered from medical records (S4 Table) and the Fisher’s exact test was used.

### Genotyping by KASP technology

To evaluate the frequency in the general Northern Portuguese population of the missense variants identified in our series of PrCa patients we used KASP technology genotyping (KBioscience, Herts, UK) in our series of 710 healthy individuals, following manufacturer’s recommendations. KASP assay primers (S6 Table) were designed using the Primer-BLAST design tool from NCBI and data were analyzed in the LightCycler 480 Software 1.5.0.

## Supporting information

S1 Fig Pedigrees of the carriers of the *CHEK2* “likely pathogenic” mutation c.349A>G. (A) Patient HPC188. (B) Patient HPC289. DCIS- Ductal carcinoma *in situ*. Representative electropherogram of the Sanger sequencing is shown.
(TIF)

S2 Fig Pedigree of the carrier of the *MSH6* in-frame mutation c.3846_3860del considered incidental finding. Note that the pedigree is the same shown in Fig 2 but here with the results of the genetic testing for the *MSH6* variant.
(TIF)

S1 Table *In silico* pathogenicity prediction scores of splicing variants. (DOCX)

S2 Table Rankscores of *in silico* pathogenicity predictors of missense variants in genes described to predispose to prostate, breast, and/or ovarian cancer and/or Lynch syndrome. (DOCX)

S3 Table Genotype frequencies observed for the missense variants in 710 healthy controls, in 504 non-prostate cancer patients and in NFE controls from ExAC. (DOCX)

S4 Table Patients’ characteristics (fulfilled criteria), mutational status and clinicopathological data. (DOCX)

S5 Table Clinicopathological associations between mutation carriers and non-carriers considering homogeneous sample groups. (DOCX)

S6 Table Primers used for Sanger sequencing and KASP genotyping. (DOCX)
