# Supplementary figures and images for: Targeted next generation sequencing identifies functionally deleterious germline mutations in novel genes in early-onset/familial prostate cancer
Source: PLoS Genet. 2018 Apr 16;14(4):e1007355. doi: 10.1371/journal.pgen.1007355 (PMC5919682; doi:10.1371/journal.pgen.1007355)

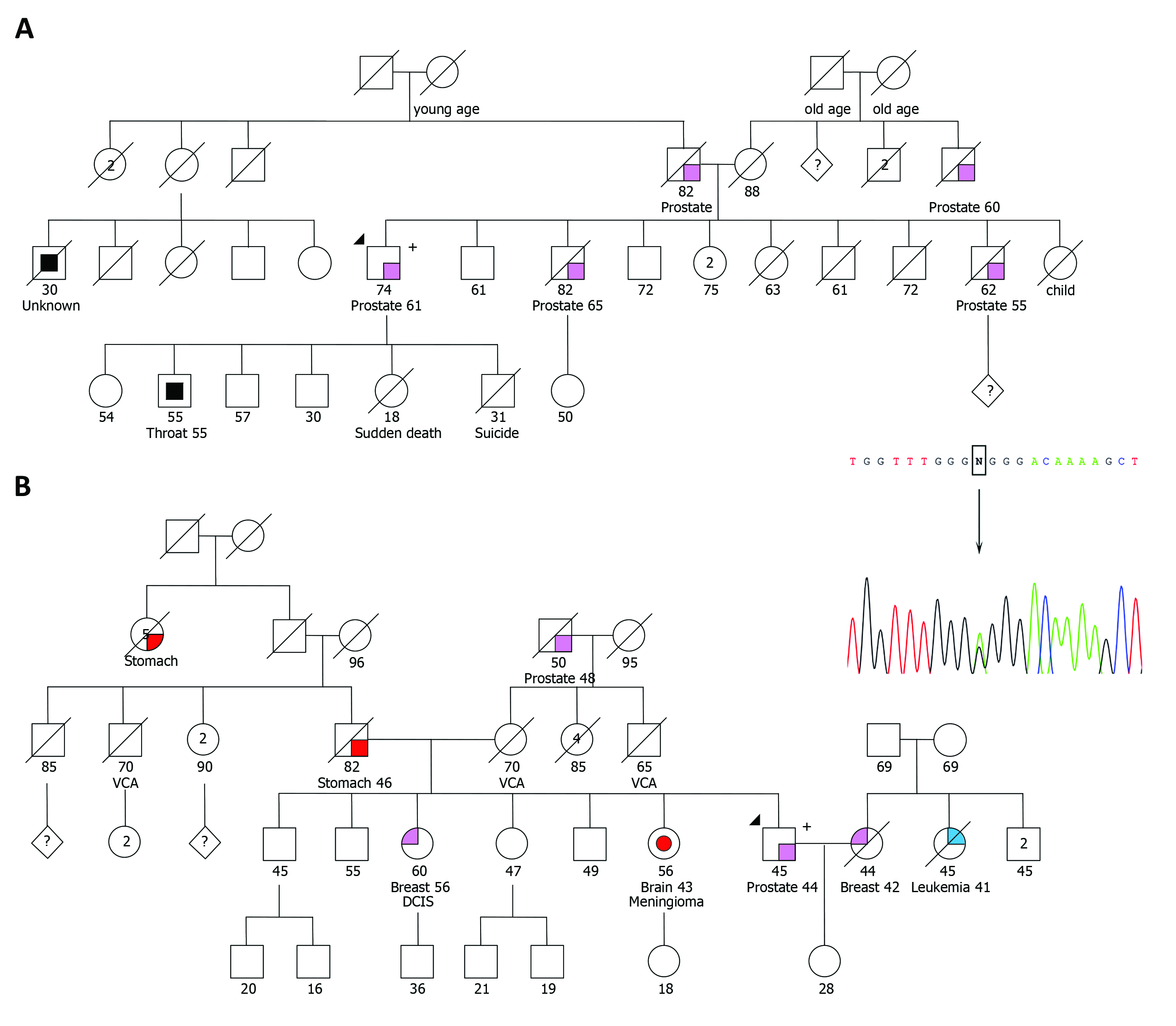

Supplement: S1 Fig — (A) Patient HPC188. (B) Patient HPC289. DCIS- Ductal carcinoma in situ. Representative electropherogram of the Sanger sequencing is shown. (TIF) [file pgen.1007355.s001.tif]

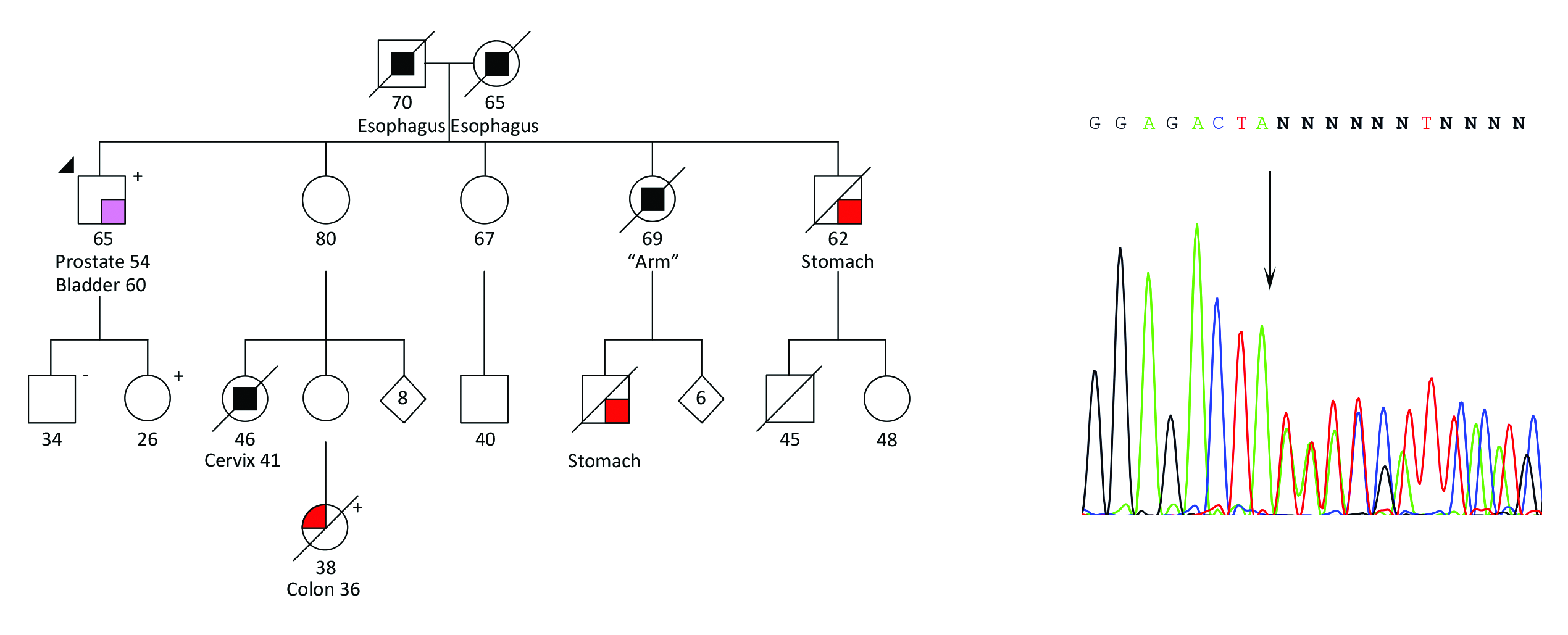

Supplement: S2 Fig — Note that the pedigree is the same shown in Fig 2 but here with the results of the genetic testing for the MSH6 variant. (TIF) [file pgen.1007355.s002.tif]
