# Supplementary material for: Targeted next generation sequencing identifies functionally deleterious germline mutations in novel genes in early-onset/familial prostate cancer
Source: PLoS Genet. 2018 Apr 16;14(4):e1007355. doi: 10.1371/journal.pgen.1007355 (PMC5919682; doi:10.1371/journal.pgen.1007355)
Supplement: S1 Table — (DOCX) [file pgen.1007355.s003.docx]

| **S1 Table.** *In silico* pathogenicity prediction scores of splicing variants. | | |
| --- | --- | --- |
| **Web-based tool** | ***CHEK2***  **c.593-1G>T** | ***FANCD2***  **c.2494+2T>C** |
| **Human Splice Finder 3.0** | Alteration of the Wt acceptor site; most probably affecting splicing. | Alteration of the Wt donor site; most probably affecting splicing. |
| **MaxEntScan** (score: Wt 🡪 Mut) |  |  |
| Maximum Entropy Model | -20.77 🡪 -30.20* | -23.79 🡪 -24.65 |
| Maximum Dependence Decomposition | -6.63 🡪 -18.53* | -15.38 🡪 -15.18 |
| First-order Markov Model | -8.34 🡪 -21.03* | -17.21 🡪 -16.68 |
| Weight Matrix Model | -10.20 🡪 -14.32* | -16.70 🡪 -16.63 |
| **NNSPLICE** | Acceptor site lost | Donor site lost |
| **NetGene2** | No donor or acceptor site predictions | Donor site lost |

Wt- wild-type allele; Mut- mutated allele; *change in the score is considered relevant (>30% difference between Wt and Mut)
