# Supplementary material for: Targeted next generation sequencing identifies functionally deleterious germline mutations in novel genes in early-onset/familial prostate cancer
Source: PLoS Genet. 2018 Apr 16;14(4):e1007355. doi: 10.1371/journal.pgen.1007355 (PMC5919682; doi:10.1371/journal.pgen.1007355)
Supplement: S2 Table — (DOCX) [file pgen.1007355.s004.docx]

| **S2 Table.** Rankscores of *in silico* pathogenicity predictors of missense variants in genes described to predispose to prostate, breast, and/or ovarian cancer and/or Lynch syndrome. | | | | | | | | | | | | | | | | | |
| --- | --- | --- | --- | --- | --- | --- | --- | --- | --- | --- | --- | --- | --- | --- | --- | --- | --- |
| **Gene** | **Variant** | **SIFT** | **PolyPhen2 HDIV** | **LRT** | **Mutation Taster** | **FATHMM** | **PROVEAN** | **CADD^[[1]](#endnote-1)^** | **Mutation Assessor** | **MetaSVM** | **MetaLR** | **VEST3** | **PhyloP^[[2]](#endnote-2)^** | **GERP++** | **Phast Cons^ii^** | **SiPhy** | ***In silico* predicted pathogenicity^[[3]](#endnote-3)^** |
| ***ATM*** | c.649A>G | 0.258 (T) | 0.013 (T) | 0.419 (T) | 0.207 (T) | 0.017 (T) | 0.125 (N) | 0.291 (T) | 0.514 (T) | 0.478 (T) | 0.020 (T) | 0.134 (T) | 0.153 | 0.356 | 0.271 | 0.390 | 0/15 |
| ***ATM*** | c.670A>G | **0.587 (D)** | **0.537 (D)** | **0.644 (D)** | **0.394 (D)** | 0.021 (T) | 0.497 (N) | **0.753 (D)** | **0.690 (M)** | 0.112 (T) | 0.061 (T) | **0.765 (P)** | **0.666** | **0.768** | **0.714** | **0.771** | 11/15 |
| ***ATM*** | c.995A>G | **0.709 (D)** | **0.899 (D)** | **0.857 (D)** | **0.708 (D)** | 0.021 (T) | **0.811 (D)** | **0.947 (P)** | **0.670 (D)** | 0.004 (T) | 0.116 (T) | **0.908 (P)** | **0.753** | **0.895** | **0.714** | **0.804** | **12/15** |
| ***ATM*** | c.1273G>T | 0.028 (T) | 0.040 (T) | 0.109 (T) | 0.101 (T) | 0.016 (T) | 0.049 (N) | 0.106 (T) | 0.116 (T) | 0.473 (T) | 0.008 (T) | 0.269 (T) | 0.182 | 0.062 | 0.204 | 0.134 | 0/15 |
| ***ATM*** | c.1595G>A | **0.773 (D)** | **0.899 (D)** | **0.644 (D)** | **0.708 (D)** | 0.757 (T) | **0.791 (D)** | **0.804 (P)** | **0.650 (D)** | **0.845 (D)** | **0.840 (D)** | **0.901 (P)** | **0.646** | **0.997** | **0.714** | **0.825** | **14/15** |
| ***ATM*** | c.1703G>T | 0.309 (T) | 0.194 (T) | 0.238 (T) | 0.101 (T) | 0.545 (T) | 0.435 (N) | 0.395 (T) | 0.575 (T) | 0.287 (T) | 0.413 (T) | 0.485 (T) | 0.263 | 0.331 | 0.350 | 0.075 | 0/15 |
| ***ATM*** | c.2494C>T | 0.214 (T) | 0.013 (T) | 0.021 (T) | 0.101 (T) | 0.018 (T) | 0.237 (N) | 0.240 (T) | 0.020 (T) | 0.463 (T) | 0.008 (T) | 0.573 (T) | 0.084 | 0.084 | 0.059 | 0.002 | 0/15 |
| ***ATM*** | c.4414T>G | **0.602 (D)** | **0.519 (D)** | **0.644 (D)** | **0.320 (D)** | 0.757 (T) | 0.514 (N) | 0.280 (T) | 0.581 (T) | 0.693 (T) | 0.705 (T) | 0.531 (T) | 0.337 | 0.109 | 0.358 | 0.171 | 4/15 |
| ***ATM*** | c.5612C>T | **0.574 (D)** | **0.452 (D)** | **0.561 (D)** | 0.228 (T) | 0.778 (T) | 0.572 (N) | 0.266 (T) | **0.683 (D)** | 0.670 (T) | 0.753 (T) | 0.350 (T) | 0.363 | 0.305 | 0.298 | 0.471 | 4/15 |
| ***ATM*** | c.5750G>A | **0.906 (D)** | **0.765 (D)** | **0.859 (D)** | **0.708 (D)** | 0.802 (T) | **0.603 (D)** | **0.641 (P)** | **0.795 (D)** | **0.895 (D)** | **0.894 (D)** | **0.799 (P)** | **0.856** | **0.978** | **0.714** | **0.903** | **14/15** |
| ***ATM*** | c.5890A>G | 0.142 (T) | **0.385 (D)** | **0.857 (D)** | **0.708 (D)** | 0.014 (T) | 0.189 (N) | 0.487 (T) | 0.509 (T) | 0.636 (T) | 0.021 (T) | 0.459 (T) | **0.748** | **0.564** | **0.714** | **0.570** | 7/15 |
| ***ATM*** | c.8560C>T | **0.906 (D)** | **0.899 (D)** | **0.857 (D)** | **0.708 (D)** | 0.783 (T) | **0.910 (D)** | **0.620 (P)** | **0.902 (D)** | **0.922 (D)** | **0.907 (D)** | **0.994 (P)** | **0.860** | **0.831** | **0.714** | **0.950** | **14/15** |
| ***BLM*** | c.43C>T | 0.507 (T) | **0.899 (D)** | 0.279 (T) | **0.708 (D)** | 0.617 (T) | 0.553 (N) | **0.974 (D)** | **0.749 (D)** | 0.748 (T) | 0.719 (T) | **0.674 (P)** | **0.573** | **0.878** | **0.714** | **0.737** | 9/15 |
| ***BLM*** | c.388A>G | 0.234 (T) | 0.040 (T) | 0.036 (T) | 0.101 (T) | 0.461 (T) | 0.277 (N) | 0.033 (T) | 0.388 (T) | 0.227 (T) | 0.338 (T) | 0.028 (T) | 0.239 | 0.304 | 0.171 | 0.063 | 0/15 |
| ***BLM*** | c.968A>G | 0.124 (T) | **0.726 (D)** | **0.539 (D)** | 0.256 (T) | 0.494 (T) | 0.313 (N) | 0.465 (T) | **0.678 (D)** | 0.555 (T) | 0.610 (T) | 0.485 (T) | 0.489 | **0.731** | **0.714** | **0.506** | 6/15 |
| ***BLM*** | c.4240T>C | **0.906 (D)** | **0.717 (D)** | **0.506 (D)** | **0.335 (D)** | 0.578 (T) | 0.484 (N) | **0.795 (D)** | **0.736 (D)** | 0.666 (T) | 0.684 (T) | 0.472 (N) | 0.627 | **0.997** | 0.352 | **0.697** | 8/15 |
| ***BRCA2*** | c.280C>T | 0.308 (T) | **0.542 (D)** | **0.493 (D)** | **0.333 (D)** | 0.013 (T) | **0.905 (D)** | **0.602 (D)** | 0.528 (T) | 0.562 (T) | 0.035 (T) | **0.717 (P)** | 0.399 | **0.624** | **0.714** | 0.437 | 8/15 |
| ***BRCA2*** | c.599C>T | **0.906 (D)** | **0.899 (D)** | **0.644 (D)** | **0.382 (D)** | 0.037 (T) | **0.831 (D)** | **0.905 (D)** | **0.670 (D)** | 0.000 (T) | 0.187 (T) | 0.037 (T) | **0.696** | **0.845** | **0.714** | **0.954** | 11/15 |
| ***BRCA2*** | c.1564G>C | 0.327 (T) | 0.352 (T) | 0.054 (T) | 0.101 (T) | 0.007 (T) | 0.451 (N) | 0.336 (T) | 0.224 (T) | 0.490 (T) | 0.007 (T) | **0.713 (P)** | 0.248 | 0.306 | 0.059 | 0.174 | 1/15 |
| ***BRCA2*** | c.3245A>G | 0.317 (T) | 0.081 (T) | 0.267 (T) | 0.101 (T) | 0.009 (T) | 0.285 (N) | 0.125 (T) | 0.432 (T) | 0.475 (T) | 0.011 (T) | 0.484 (T) | 0.256 | 0.347 | 0.247 | 0.162 | 0/15 |
| ***BRCA2*** | c.6988A>G | 0.009 (T) | 0.040 (T) | 0.098 (T) | 0.101 (T) | 0.745 (T) | 0.067 (N) | 0.006 (T) | 0.023 (T) | 0.466 (T) | 0.419 (T) | 0.497 (T) | 0.036 | 0.026 | 0.059 | **0.608** | 0/15 |
| ***BRCA2*** | c.7265G>A | 0.268 (T) | 0.309 (T) | 0.011 (T) | 0.101 (T) | 0.779 (T) | 0.397 (N) | 0.200 (T) | 0.575 (T) | 0.528 (T) | 0.657 (T) | 0.655 (T) | 0.038 | 0.070 | 0.059 | 0.193 | 0/15 |
| ***BRCA2*** | c.8902A>G | 0.120 (T) | 0.040 (T) | 0.266 (T) | 0.101 (T) | 0.716 (T) | 0.228 (N) | 0.015 (T) | 0.477 (T) | 0.333 (T) | 0.536 (T) | 0.246 (T) | 0.098 | 0.016 | 0.127 | 0.489 | 0/15 |
| ***BRCA2*** | c.9011A>G | 0.088 (T) | 0.013 (T) | 0.353 (T) | 0.101 (T) | 0.727 (T) | 0.184 (N) | N/A | 0.610 (T) | 0.321 (T) | 0.555 (T) | 0.485 (T) | 0.244 | 0.103 | 0.230 | 0.026 | 0/15 |
| ***BRIP1*** | c.3178G>C | 0.116 (T) | 0.040 (T) | 0.119 (T) | 0.101 (T) | 0.737 (T) | 0.127 (N) | 0.124 (T) | 0.191 (T) | 0.292 (T) | 0.521 (T) | 0.035 (T) | 0.141 | 0.208 | 0.056 | 0.054 | 0/15 |
| ***BRIP1*** | c.847T>C | **0.906 (D)** | **0.899 (D)** | **0.857 (D)** | **0.708 (D)** | **0.929 (D)** | **0.994 (D)** | **0.890 (P)** | **0.997 (D)** | **0.987 (D)** | **0.975 (D)** | **0.961 (P)** | **0.910** | **0.747** | **0.714** | **0.695** | **15/15** |
| ***CHEK2*** | c.1036C>T | **0.906 (D)** | **0.899 (D)** | **0.857 (D)** | **0.708 (D)** | 0.657 (T) | **0.950 (D)** | **0.939 (D)** | **0.882 (D)** | 0.825 (T) | 0.799 (T) | **0.976 (P)** | 0.474 | 0.400 | 0.714 | 0.424 | 8/15 |
| ***CHEK2*** | c.953G>A | N/A | N/A | N/A | **0.807 (D)** | N/A | N/A | **0.741 (D)** | 0.568 (T) | 0.350 (T) | 0.432 (T) | N/A | 0.437 | **0.831** | 0.298 | **0.521** | 4/9 |
| ***CHEK2*** | c.715G>A | 0.364 (T) | **0.658 (D)** | **0.644 (D)** | **0.708 (D)** | **0.824 (D)** | 0.451 (N) | 0.501 (T) | 0.056 (T) | **0.763 (A)** | **0.730 (A)** | **0.924 (P)** | **0.507** | **0.741** | **0.714** | **0.767** | 11/15 |
| ***CHEK2*** | c.695G>T | **0.906 (D)** | **0.899 (D)** | **0.857 (D)** | **0.708 (D)** | 0.807 (T) | **0.975 (D)** | 0.487 (N) | **0.973 (D)** | **0.954 (D)** | **0.933 (D)** | **0.984 (P)** | **0.617** | **0.709** | **0.714** | **0.744** | **13/15** |
| ***CHEK2*** | c.349A>G | **0.906 (D)** | **0.899 (D)** | **0.857 (D)** | **0.708 (D)** | **0.975 (D)** | **0.918 (D)** | **0.852 (P)** | **0.983 (D)** | **0.989 (D)** | **0.990 (D)** | **0.931 (P)** | 0.360 | **0.619** | **0.714** | **0.564** | **14/15** |
| ***MSH2*** | c.1571G>A | **0.906 (D)** | **0.899 (D)** | **0.857 (D)** | **0.708 (D)** | **0.902 (D)** | **0.812 (D)** | **0.962 (P)** | **0.894 (D)** | **0.966 (D)** | **0.959 (D)** | **0.893 (P)** | **0.930** | **0.712** | **0.714** | **0.722** | **15/15** |
| ***MSH6*** | c.1729C>T | **0.906 (D)** | **0.460 (D)** | **0.857 (D)** | **0.708 (D)** | **0.880 (D)** | **0.827 (D)** | **0.568 (P)** | **0.660 (D)** | **0.875 (D)** | **0.871 (D)** | **0.930 (P)** | **0.706** | **0.677** | **0.714** | **0.918** | **15/15** |
| ***NBN*** | c.1999T>C | 0.067 (T) | 0.081 (T) | 0.064 (T) | 0.101 (T) | 0.588 (T) | 0.068 (N) | 0.027 (T) | 0.271 (T) | 0.173 (T) | 0.384 (T) | 0.042 (T) | 0.275 | 0.152 | 0.236 | 0.070 | 0/15 |
| ***PALB2*** | c.3272A>G | 0.164 (T) | **0.450 (D)** | 0.293 (T) | 0.221 (T) | 0.141 (T) | 0.369 (N) | 0.464 (T) | 0.422 (T) | 0.121 (T) | 0.119 (T) | 0.284 (T) | 0.291 | **0.631** | **0.714** | 0.218 | 3/15 |
| ***PALB2*** | c.3251C>T | 0.087 (T) | 0.094 (T) | 0.033 (T) | 0.101 (T) | 0.163 (T) | 0.502 (N) | 0.216 (T) | 0.019 (T) | 0.301 (T) | 0.068 (T) | 0.040 (T) | 0.342 | 0.163 | 0.266 | 0.205 | 3/15 |
| ***PALB2*** | c.1751A>G | 0.442 (T) | 0.230 (T) | 0.241 (T) | 0.101 (T) | 0.193 (T) | **0.607 (D)** | 0.174 (T) | **0.627 (D)** | 0.205 (T) | 0.206 (T) | 0.548 (T) | 0.204 | 0.398 | 0.223 | 0.261 | 2/15 |
| ***PALB2*** | c.932A>G | 0.399 (T) | 0.264 (T) | 0.068 (T) | 0.101 (T) | 0.150 (T) | 0.084 (N) | 0.027 (T) | 0.224 (T) | 0.322 (T) | 0.131 (T) | 0.053 (T) | 0.111 | 0.325 | 0.059 | 0.203 | 0/15 |
| ***PMS2*** | c.2182A>G | 0.081 (T) | 0.151 (T) | **0.456 (D)** | **0.708 (D)** | 0.743 (T) | 0.349 (N) | 0.296 (T) | 0.453 (T) | 0.510 (T) | 0.599 (T) | 0.092 (T) | 0.365 | 0.382 | **0.714** | 0.288 | 3/15 |
| ***PMS2*** | c.857A>G | 0.466 (T) | 0.306 (T) | 0.320 (T) | **0.708 (D)** | **0.852 (D)** | **0.886 (D)** | 0.303 (T) | **0.854 (D)** | **0.883 (D)** | **0.885 (D)** | **0.795 (P)** | **0.839** | **0.937** | **0.714** | **0.827** | 11/15 |
| ***TP53*** | c.869G>A | 0.295 (T) | 0.068 (T) | 0.136 (T) | **0.708 (D)** | **0.998 (D)** | 0.504 (N) | 0.362 (T) | 0.445 (T) | **0.960 (D)** | **0.986 (D)** | **0.701 (P)** | 0.045 | 0.095 | 0.059 | 0.400 | 5/15 |
| ***TP53*** | c.839G>A | **0.906 (D)** | **0.691 (D)** | **0.857 (D)** | **0.708 (D)** | **0.999 (D)** | **0.601 (D)** | **0.957 (P)** | **0.926 (D)** | **0.964 (D)** | **0.999 (D)** | **0.957 (P)** | **0.826** | **0.701** | 0.344 | **0.813** | **14/15** |

1. Notes: For each tool, predicted functional impact is shown in brackets: P- possibly damaging/deleterious, D- probably damaging/deleterious and T- tolerated. The predicted pathogenicity of the non-prostate cancer related *MSH2* and *MSH6* variants is also shown. N/A- not available.

   We considered variants with a CADD rankscore above 0.51 as possibly damaging (P) and below as neutral (N) (Buitrago *et al*, 2015). [↑](#endnote-ref-1)
2. Among vertebrates (100way). [↑](#endnote-ref-2)
3. Fraction of functional predictors where the variant is expected to be pathogenic (highlighted in bold letters), which includes the classifications D and P, and the conservation predictors with score >0.5, among the 15 tools tested. [↑](#endnote-ref-3)
