# Supplementary material for: Targeted next generation sequencing identifies functionally deleterious germline mutations in novel genes in early-onset/familial prostate cancer
Source: PLoS Genet. 2018 Apr 16;14(4):e1007355. doi: 10.1371/journal.pgen.1007355 (PMC5919682; doi:10.1371/journal.pgen.1007355)
Supplement: S3 Table — (DOCX) [file pgen.1007355.s005.docx]

| **S3 Table.** Genotype frequencies observed for the missense variants in 710 healthy controls, in 504 non-prostate cancer patients and in NFE controls from ExAC. | | | | | | | | |
| --- | --- | --- | --- | --- | --- | --- | --- | --- |
| **Gene** | **Variant** | **Frequency in Controls** | **Frequency in other cancers** | **Frequency in NFE controls** | **Frequency in PrCa-patients** | **PrCa-patients**  ***vs.* Controls***  (*P value*) | **PrCa-patients**  ***vs.* other cancers***  (*P value*) | **PrCa-patients**  ***vs.* NFE controls****  (*P value*) |
| ***ATM*** | c.995A>G | 0 | 0 | ND | 0.0083 | 0.146 | 0.194 | NA |
| ***ATM*** | c.1595G>A | 0.0028 | 0.0119 | 9/33308 | 0.0165 | 0.103 | 0.656 | <0.0001 |
| ***ATM*** | c.5750G>A | 0 | 0 | 1/32529 | 0.0083 | 0.146 | 0.194 | <0.0001 |
| ***ATM*** | c.8560C>T | 0.0028 | 0.0060 | 13/33352 | 0.0248 | **0.024** | 0.090 | <0.0001 |
| ***BRIP1*** | c.847T>C | 0 | 0.0020 | ND | 0.0083 | 0.146 | 0.350 | NA |
| ***CHEK2*** | c.349A>G | 0.0014 | 0.0060 | 13/33315 | 0.0165 | **0.057** | 0.249 | <0.0001 |
| ***CHEK2*** | c.695G>T | 0 | 0.0020 | 0/33338 | 0.0083 | 0.146 | 0.350 | <0.0001 |
| ***TP53*** | c.839G>A | 0 | 0 | ND | 0.0083 | 0.146 | 0.194 | NA |
| *Fisher’s exact test; **Chi-square test with Yates correction; N/D- not described; N/A- not assessed; NFE- Non-Finnish Europeans. | | | | | | | | |
