# Supplementary material for: Targeted next generation sequencing identifies functionally deleterious germline mutations in novel genes in early-onset/familial prostate cancer
Source: PLoS Genet. 2018 Apr 16;14(4):e1007355. doi: 10.1371/journal.pgen.1007355 (PMC5919682; doi:10.1371/journal.pgen.1007355)
Supplement: S4 Table — (DOCX) [file pgen.1007355.s006.docx]

**S4 Table.** Patients’ characteristics (fulfilled criteria), carrying mutations and clinicopathological data.

| **Sample** | **Age at diagnosis** | **Fulfilled criteria** | **Mutation consequence** | **Mutated Gene(s)^[[1]](#endnote-1)^** | **PSA at Diagnosis** | **Gleason Score^[[2]](#endnote-2)^** | **T-Stage^[[3]](#endnote-3)^** | **N-Stage** | **M-Stage** | **Treatment** |
| --- | --- | --- | --- | --- | --- | --- | --- | --- | --- | --- |
| HPC3 | 53 | A3 | Missense | *ATM* | 6.61 | 9 (4+5) | pT3b | N0 | M0 | PT+RT+ADT |
| HPC16 | 51 | B1 | N/A | N/A | 4.93 | 6 (3+3) | pT2c | Nx | Mx | PT |
| HPC17 | 52 | B1 | N/A | N/A | 9.60 | 6 (3+3) | pT2 | N0 | M0 | PT |
| HPC20 | 55 | B1 | Missense | *ATM* | 4.80 | 6 (3+3) | pT2a | N0 | M0 | PT+RT |
| HPC22 | 54 | B1 | N/A | N/A | 26.00 | 7 (4+3) | pT2c | N0 | M0 | PT |
| HPC23 | 46 | B1 | N/A | N/A | 5.60 | 7 (3+4) | pT2c | Nx | Mx | PT |
| HPC35 | 55 | B1 | N/A | N/A | 9.60 | 7 (4+3) | pT3a | Nx | Mx | PT+RT+ADT |
| HPC38 | 49 | B1 | N/A | N/A | 3.80 | 10 (5+5) | pT3b | Nx | Mx | PT+RT |
| HPC41 | 51 | B2 | N/A | N/A | 5.38 | 6 (3+3)* | pT3b* | N0 | M0 | BT+PT+ADT |
| HPC43 | 52 | A3, B1 | N/A | N/A | 5.91 | 6 (3+3) | pT2 | N0 | M0 | PT+ADT |
| HPC44 | 51 | B1 | N/A | N/A | 6.77 | 7 (3+4) | pT2c | N0 | Mx | PT |
| HPC46 | 52 | A2, A3 | N/A | N/A | 6.49 | 6 (3+3) | pT2c | N0 | M0 | PT |
| HPC55 | 46 | B1 | N/A | N/A | 9.90 | 6 (3+3) | pT3a | Nx | M0 | PT |
| HPC59 | 53 | B1, B2 | N/A | N/A | 7.40 | 7 (3+4) | pT3a | N0 | M0 | PT+RT |
| HPC60 | 53 | A2, A3, B1 | N/A | N/A | 9.41 | 7 (4+3) | pT3a | Nx | Mx | PT |
| HPC66 | 53 | B1 | N/A | N/A | 5.75 | 7 (4+3) | pT3a | Nx | Mx | PT |
| HPC71 | 52 | B1 | Frameshift | *TSC2*^#^ | 3.81 | 5 (3+2) | pT2 | Nx | M0 | PT+ADT |
| HPC74 | 54 | B1 | N/A | N/A | 8.69 | 7 (4+3) | pT3a | N0 | Mx | PT+RT |
| HPC75 | 53 | B1 | N/A | N/A | 7.10 | 7 (3+4) | pT3a | Nx | M0 | PT+RT |
| HPC81 | 62 | A1 | N/A | N/A | 7.60 | 7 (3+4) | pT3a | N0 | M0 | PT |
| HPC82 | 68 | A2 | N/A | N/A | 9.20 | 7 (3+4) | pT3b | Nx | M0 | PT |
| HPC85 | 40 | B1 | N/A | N/A | 6.40 | 7 (3+4) | pT3a | Nx | Mx | PT+ADT |
| HPC87 | 55 | B1 | N/A | N/A | 85.00 | 8 (4+4)* | cT3 | N0 | M0 | PT+RT+ADT |
| HPC88 | 79 | B3 | N/A | N/A | 6.00 | 7 (3+4)* | U | Nx | Mx | AS |
| HPC89 | 55 | B2 | Missense | *CHEK2* | 12.70 | U | pT2c* | Nx | Mx | PT+RT+ADT |
| HPC90 | 54 | B1 | N/A | N/A | 11.80 | 6* | cT3a | Nx | Mx | RT+ADT |
| HPC93 | 55 | B1 | N/A | N/A | 5.80 | 7 (3+4)* | cT2a | Nx | Mx | BT |
| HPC97 | 50 | B1 | N/A | N/A | 108.90 | 7 (4+3)* | cT4 | Nx | Mx | ADT |
| HPC100 | 68 | B3 | N/A | N/A | 5.40 | 7 (4+3) | pT3a | Nx | Mx | PT |
| HPC113 | 62 | B2 | N/A | N/A | U | 7 (3+4) | pT3b | Nx | M0 | PT+ADT |
| HPC115 | 52 | A2, A3 | N/A | N/A | 6.00 | 6 (2+4) | pT2 | Nx | M0 | PT |
| HPC116 | 63 | B3 | N/A | N/A | 8.90 | 6 (3+3) | pT2c | N0 | M0 | PT |
| HPC118 | 62 | A2 | Missense | *BRIP1* | 9.00 | 6 (3+3)* | cT3a | N0 | M0 | PT+RT+ADT |
| HPC119 | 55 | A1, A2, A3 | N/A | N/A | 10.51 | 6 (3+3)* | cT3a | N0 | M0 | RT+ADT |
| HPC120 | 54 | A2, A3, B1 | N/A | N/A | 12.40 | 7 (3+4)* | cT2c | N0 | M0 | RT+ADT |
| HPC121 | 51 | A1, A2, A3 | N/A | N/A | 5.12 | 5 (3+2) | pT2b | Nx | M0 | PT+RT+ADT |
| HPC122 | 55 | A1, A2, A3 | N/A | N/A | 6.10 | 7 (4+3) | pT3a | Nx | Mx | PT+RT+ADT |
| HPC125 | 62 | A2 | N/A | N/A | 11.15 | 8 (4+4)* | cT2 | Nx | Mx | RT+ADT |
| HPC131 | 54 | B1 | N/A | N/A | 9.50 | 6 (3+3)* | cT1c | N0 | M0 | RT |
| HPC134 | 55 | A1, A2, A3, B1 | N/A | N/A | 11.40 | 6 (3+3)* | cT2 | N0 | M1 | RT |
| HPC137 | 54 | B1 | N/A | N/A | 4.25 | 6 (3+3)* | cT1c | N0 | M0 | BT |
| HPC144 | 64 | A1, A2 | N/A | N/A | 34.40 | 5 (2+3)* | cT2 | N0 | M0 | RT+ADT |
| HPC145 | 64 | A2, B2 | N/A | N/A | 10.70 | 5 (3+2) | pT3a | N0 | M0 | PT+RT |
| HPC146 | 59 | A2, A3 | N/A | N/A | 4.45 | 6 (3+3)* | cT2b | Nx | M0 | BT |
| HPC147 | 57 | A3 | N/A | N/A | 4.98 | 6 (3+3)* | cT1c | N0 | M0 | RT+ADT |
| HPC150 | 66 | A2 | Frameshift | *FANCI** | 35.80 | 6 (3+3)* | U | Nx | Mx | PT+RT+ADT |
| HPC152 | 58 | A3 | N/A | N/A | 7.90 | 7 (3+4) | pT2c | Nx | Mx | PT |
| HPC154 | 53 | B1 | N/A | N/A | 4.00 | 7 (3+4) | pT2a | Nx | M0 | PT+RT |
| HPC155 | 54 | B2 | N/A | N/A | 6.50 | 7 (3+4)* | U | Nx | Mx | PT+ADT |
| HPC156 | 50 | B1, B2 | N/A | N/A | 16.28 | 7 (3+4) | pT3a | Nx | M0 | PT+RT+ADT |
| HPC158 | 53 | B1 | N/A | N/A | 7.60 | 7 (4+3)* | cT3 | N0 | M0 | PT+RT+ADT |
| HPC160 | 55 | B3 | N/A | N/A | 13.00 | 7 (3+4) | pT2c | Nx | M0 | PT+ADT |
| HPC165 | 59 | A1, B2 | N/A | N/A | 11.90 | 7 (3+4) | pT3a | N0 | M0 | PT+RT |
| HPC167 | 60 | B2 | Missense | *ATM* | 4.50 | 6 (3+3)* | cT2 | N0 | M0 | RT+ADT |
| HPC168 | 62 | B3 | N/A | N/A | 9.40 | 3* | cT3 | N0 | M0 | RT |
| HPC171 | 64 | A1, A2 | N/A | N/A | 5.50 | 7 (3+4) | pT3a | Nx | M0 | PT |
| HPC177 | 63 | A1, A2 | Nonsense | *ATM** | 7.22 | 6 (4+2)* | cT3a | Nx | M0 | RT+ADT |
| HPC180 | 53 | A2, A3, B1 | N/A | N/A | 8.52 | 7 (3+4)* | cT1c | Nx | Mx | RT |
| HPC186 | 54 | B1, B2 | Frameshift Missense  Missense | *RAD51C**  *ATM*  *MSH6*^#^ | 24.30 | 6 (3+3)* | cT2b | N0 | M0 | RT+ADT |
| HPC188 | 61 | A1, A2 | Missense | *CHEK2** | 33.29 | 5 (3+2)* | cT3 | N0 | Mx | RT+ADT |
| HPC207 | 55 | B1 | N/A | N/A | 6.60 | 7 (3+4) | pT2b | N0 | M0 | PT |
| HPC221 | 47 | B1 | N/A | N/A | 11.07 | 7 (3+4) | pT3a | Nx | Mx | PT |
| HPC222 | 61 | A2 | N/A | N/A | U | 7 (3+4) | pT2c | N0 | M0 | PT+ADT |
| HPC224 | 56 | A1, A2, A3 | N/A | N/A | 5.20 | 6 (3+3)* | cT1c | N0 | M0 | RT+ADT |
| HPC230 | 60 | A1 | N/A | N/A | 5.00 | 9 (4+5) | pT3a | Nx | Mx | PT+ADT |
| HPC232 | 61 | B1 | N/A | N/A | 8.80 | 6 (3+3)* | cT3a | N0 | M0 | RT+ADT |
| HPC235 | 58 | A1, A2, A3 | N/A | N/A | 4.94 | 7 (3+4) | pT2c | Nx | Mx | PT |
| HPC238 | 60 | A1, A2, A3 | Missense | *ATM* | 5.99 | 7 (3+4) | pT2a | Nx | Mx | PT |
| HPC240 | 54 | B2 | N/A | N/A | 7.12 | 6 (3+3) | pT2c | N0 | M0 | PT |
| HPC245 | 64 | A1, A2 | N/A | N/A | 5.13 | 6 (3+3)* | cT1c | Nx | M0 | RT |
| HPC247 | 61 | B2 | N/A | N/A | 7.90 | 7 (3+4)* | cT2b | Nx | M0 | RT+ADT |
| HPC249 | 57 | A1, A2, A3 | N/A | N/A | 6.30 | 6 (3+3) | pT2c | N0 | M0 | PT |
| HPC252 | 60 | A3 | N/A | N/A | 470.88 | 7 (3+4)* | cT4 | Nx | M1 | ADT |
| HPC262 | 52 | A3 | N/A | N/A | 331.00 | 7 (3+4)* | cT4 | N0 | M1 | ADT+CT |
| HPC268 | 61 | A2 | N/A | N/A | 3.50 | 6 (3+3) | pT2c | N0 | M0 | PT+RT |
| HPC269 | 64 | B3 | N/A | N/A | 6.50 | 7 (4+3)* | cT1c | Nx | M0 | RT+ADT |
| HPC275 | 53 | B1 | N/A | N/A | 15.32 | 9 (4+5)* | cT4 | Nx | M0 | RT+ADT |
| HPC279 | 51 | B1 | N/A | N/A | 4.09 | 6 (3+3)* | cT2a | Nx | Mx | PT |
| HPC283 | 60 | A2, A3 | N/A | N/A | 8.00 | 7 (4+3) | pT2c | Nx | M0 | PT+ADT |
| HPC286 | 65 | A1 | N/A | N/A | 7.89 | 7 (3+4) | pT3a | Nx | M0 | PT |
| HPC289 | 44 | B1, B3 | Missense | *CHEK2** | 12.00 | 8 (4+4) | pT2b | Nx | Mx | PT |
| HPC294 | 55 | B2 | N/A | N/A | 8.49 | 6 (3+3)* | cT1c | N0 | M0 | BT |
| HPC300 | 56 | B2 | N/A | N/A | 6.40 | 9 (4+5) | pT2a | Nx | Mx | PT |
| HPC301 | 64 | A1 | N/A | N/A | 23.70 | 6 (3+3) | pT2 | Nx | Mx | PT |
| HPC306 | 57 | B2 | N/A | N/A | 9.41 | 7 (4+3)* | cT2a | N0 | M0 | RT |
| HPC307 | 52 | B1 | N/A | N/A | 6.76 | 6 (3+3)* | cT1c | Nx | M0 | BT |
| HPC332 | 54 | A3, B1 | Missense Missense | *ATM*  *MSH6*^#^ | 5952.00 | 6 (3+3)* | cT1c | N0 | M0 | BT |
| HPC335 | 52 | A2, A3 | N/A | N/A | 7.25 | 6 (3+3)* | cT1c | Nx | M0 | PT |
| HPC338 | 54 | B2 | N/A | N/A | 6.30 | 7 (3+4)* | cT1c | N0 | M0 | RT |
| HPC347 | 63 | B2 | N/A | N/A | 9.20 | 6 (3+3)* | cT1c | N0 | M0 | BT |
| HPC358 | 49 | B1 | N/A | N/A | 8.00 | 5 (2+3)* | pT3b* | Nx | M0 | BT+PT |
| HPC362 | 52 | B2 | N/A | N/A | 7.42 | 6 (3+3) | pT2c | N0 | M0 | PT+RT |
| HPC363 | 52 | B1 | N/A | N/A | 5.20 | 7 (3+4) | pT3a | N0 | Mx | PT+RT |
| HPC371 | 65 | B3 | Missense | *MSH2*^#^ | 11.95 | 8 (4+4) | pT3a | Nx | M0 | PT |
| HPC373 | 54 | B2 | N/A | N/A | 15.65 | 9 (5+4) | pT3b | Nx | Mx | PT+ADT |
| HPC374 | 63 | B2 | N/A | N/A | 5.78 | 7 (3+4)* | cT2 | Nx | M0 | ADT |
| HPC381 | 60 | A2, A3 | N/A | N/A | 7.68 | 6 (3+3) | pT2 | N0 | M0 | PT |
| HPC389 | 54 | B1 | N/A | N/A | 36.00 | 9 (4+5) | pT3b | N0 | M0 | PT+ADT |
| HPC391 | 65 | B2 | N/A | N/A | 4.70 | 7 (3+4) | pT2c | N0 | M0 | PT |
| HPC394 | 62 | A1, A2, B2 | Missense | *TP53* | 0.23 | 7 (4+3) | pT3a | Nx | Mx | PT+ADT |
| HPC395 | 63 | B3 | Splicing | *CHEK2** | 6.20 | 7 (4+3) | pT3a | N0 | M0 | PT+ADT |
| HPC400 | 51 | B1 | Missense | *ATM* | 5.10 | 8 (5+3) | pT2c | N0 | M0 | PT |
| HPC404 | 54 | B2 | N/A | N/A | 12.00 | 6 (3+3) | pT2c | Nx | Mx | PT |
| HPC416 | 48 | B1 | N/A | N/A | 7.94 | 6 (3+3) | pT3a | Nx | M0 | PT+RT |
| HPC419 | 55 | B2 | N/A | N/A | 13.70 | 6 (3+3) | pT2b | Nx | M0 | PT |
| HPC421 | 60 | B2 | Nonsense | *CEP57** | 11.40 | 6 (3+3) | pT3a | Nx | Mx | PT+RT |
| HPC422 | 64 | B2 | N/A | N/A | 11.85 | 7 (4+3) | pT2c | Nx | M0 | PT |
| HPC437 | 60 | A3 | N/A | N/A | 14.86 | 5 (2+3)* | cT2a | N0 | M0 | RT+ADT |
| HPC440 | 63 | B2 | N/A | N/A | 10.90 | 7 (3+4) | pT3a | Nx | Mx | PT |
| HPC446 | 65 | B3 | N/A | N/A | 15.00 | 7 (3+4) | pT3b | N0 | M0 | PT+RT |
| HPC447 | 59 | B3 | Splicing | *FANCD2** | 7.00 | 5 (2+3)* | cT1c | N0 | M0 | BT |
| HPC453 | 58 | B2 | N/A | N/A | 4.14 | 5 (3+2)* | cT1c | Nx | M0 | BT |
| HPC455 | 63 | B2 | Frameshift | *RECQL4** | 9.10 | 7 (3+4)* | cT2a | N0 | M0 | AS |
| HPC458 | 63 | B3 | N/A | N/A | 8.44 | 7 (3+4) | pT3a | Nx | M0 | PT |
| HPC461 | 58 | A2, A3 | N/A | N/A | 8.79 | 8 (3+5) | pT2c | Nx | Mx | PT+RT |
| HPC463 | 59 | B2 | N/A | N/A | 3.21 | 6 (3+3)* | U | N0 | M0 | RT |
| HPC478 | 54 | A2, A3, B1 | N/A | N/A | 8.00 | 5 (3+2)* | cT2 | N0 | M0 | RT |
| HPC499 | 59 | B3 | N/A | N/A | 3.00 | 6 (3+3) | pT3a | N0 | M0 | PT+RT+ADT |
| HPC501 | 51 | B1 | N/A | N/A | 4.88 | 7 (3+4) | pT2c | N0 | M0 | PT+RT |
| HPC530 | 69 | B3 | N/A | N/A | U | 7 (4+3)* | U | Nx | Mx | RT+ADT |
| HPC531 | 53 | B2, B3 | N/A | N/A | 4.25 | 7 (3+4) | pT2c | Nx | Mx | PT |

1. variants considered pathogenic are marked by an asterisk and non-PrCa associated variants are marked by a hashtag. [↑](#endnote-ref-1)
2. information obtained from biopsy is marked by an asterisk. [↑](#endnote-ref-2)
3. information obtained after brachytherapy is marked by and asterisk.

   ADT- androgen deprivation therapy; AS- active surveillance; BT- brachytherapy; CT- chemotherapy; PT- prostatectomy; RT- radiation therapy; U- unknown; N/A- not applicable [↑](#endnote-ref-3)
