# Supplementary material for: Targeted next generation sequencing identifies functionally deleterious germline mutations in novel genes in early-onset/familial prostate cancer
Source: PLoS Genet. 2018 Apr 16;14(4):e1007355. doi: 10.1371/journal.pgen.1007355 (PMC5919682; doi:10.1371/journal.pgen.1007355)
Supplement: S5 Table — (DOCX) [file pgen.1007355.s007.docx]

| **S5 Table.** Clinicopathological associations between mutation carriers and non-carriers considering homogeneous sample groups. | | | | | | | | | | | |
| --- | --- | --- | --- | --- | --- | --- | --- | --- | --- | --- | --- |
| **Parameter** | | **All cases** | | ***P value*** | **A criteria** | | ***P value*** | **Early-Onset**^i^ | | ***P value*** |  |
|  |  | Carriers | Non-carriers |  | Carriers | Non-carriers |  | Carriers | Non-carriers |  |  |
| **PSA at diagnosis** | |  |  |  |  |  |  |  |  |  |  |
|  | <10 | 11 | 73 | NS | 5 | 25 | NS | 3 | 41 | NS |  |
|  | >10 | 7 | 27 |  | 3 | 11 |  | 4 | 16 |  |  |
| **Gleason Score** | |  |  |  |  |  |  |  |  |  |  |
|  | GS≤7(3+4) | 12 | 79 | NS | 6 | 31 | NS | 3 | 44 | NS |  |
|  | GS≥7(4+3) | 5 | 24 |  | 2 | 6 |  | 3 | 13 |  |  |
| **Tumor stage** | |  |  |  |  |  |  |  |  |  |  |
|  | c/pT≤T2 | 9 | 57 | NS | 2 | 25 | NS | 5 | 31 | NS |  |
|  | c/pT≥T3 | 7 | 40 |  | 5 | 12 |  | 1 | 23 |  |  |
| NS- not significant (*P* value>0.05; Fisher’s exact test).  ^i^ cases diagnosed at the age of ≤55. | | | | | | | | | | | |
