# Supplementary material for: Targeted next generation sequencing identifies functionally deleterious germline mutations in novel genes in early-onset/familial prostate cancer
Source: PLoS Genet. 2018 Apr 16;14(4):e1007355. doi: 10.1371/journal.pgen.1007355 (PMC5919682; doi:10.1371/journal.pgen.1007355)
Supplement: S6 Table — (DOCX) [file pgen.1007355.s008.docx]

| **S6 Table.** Primers used for Sanger sequencing and KASP genotyping. | |
| --- | --- |
| **Primer Name**^i^ | **5'🡪3' Sequence** |
| ***Sanger sequencing primers*** | |
| ATM-Ex6-F | TACTTCAGGCTCTATCTGAAACC |
| ATM-Ex6-R | TAGTTCTGTTATGATGGATCAATG |
| ATM-Ex8-F | GAAGGTTGGACCAGGTGTCT |
| ATM-Ex8-R | ACATGACCTACTTACTGTACCTGG |
| ATM-Ex10-F | ACCTAACTGTGAGCTGTCTCC |
| ATM-Ex10-R | CGGCCAAACAAGAAAAGCATC |
| ATM-Ex20-F | GTAAATGATTTGTGGATAAACCTG |
| ATM-Ex20-R | GAAGAAATCACTGATGTGGATAC |
| ATM-Ex28-F | TCTGGACTGTGATATGTCATTTG |
| ATM-Ex28-R | CTAAAGTGTCACAAGATTCTGTTC |
| ATM-Ex38-F | GCAAGAATGCCTGGGACTGA |
| ATM-Ex38-R | GGAGCCAAGAAGGCTGCATA |
| ATM-Ex50-F | TTGTAGTTCTGTTAAAGTTCATGG |
| ATM-Ex50-R | CAAGTGCTAGGAATACAAAGAGG |
| ATM-Ex56-F | TGAGTGCCCTTTGCTATTCTCAG |
| ATM-Ex56-R | TGTTTTTGGTGAACTAACAGAAGT |
| ATM-Ex58-F | TCAACCAGTTTTCCGTTACTTCTG |
| ATM-Ex58-R | CCTGCCAAACAACAAAGTGCT |
| BRIP1-Ex7-F | CCTTTCTTACAGCCCCCTGG |
| BRIP1-Ex7-R | AGCAGTTAATTTGATTTTCCGAAGT |
| CHEK2-Ex3-F | TGCCTTCTTAGGCTATTTTCCT |
| CHEK2-Ex3-R | AACCATATTCTGTAAGGACAGGAC |
| CHEK2-Ex5-F | ACCCGTATGGTGATACTGATGG |
| CHEK2-Ex5-R | TACCTTCCAAGAGTTTTTGACATGA |
| CHEK2-Ex6-F | ACAGGGACAATGGAAAGGCTG |
| CHEK2-Ex6-R | ATCTAAGCAGGGGGTTATTCCTG |
| CEP57-Ex7-F | AATGTGTTCCACGCTGAAGAAG |
| CEP57-Ex7-R | TTGCCTGATTTGGTTCTGTCT |
| FANCD2-Ex26-F | ACCACGTTGTTGAGGACAGTT |
| FANCD2-Ex26-R | GCTAAGTGCTTTATTGCCTCAC |
| FANCI-Ex4-F | AGCACCGTAGTAATCAGTCGT |
| FANCI-Ex4-R | AACCCTCAAAAACCTACCCCC |
| RAD51C-Ex6-F | CAAAGAGACTCACCTAATTTTCTTACATTTTGT |
| RAD51C-Ex6-R | ACCAGTGAACAAGACAAATACAGTCTGC |
| RECQL4-Ex15-F | CACACATGGTCCCATCCCACTGA |
| RECQL4-Ex15-R | TATTGGGAGTGCCCGCTCATGG |
| TSC2-Ex26-F | TTTTCTGTCTCTTCCCCGCT |
| TSC2-Ex26-R | ACCCGACAGTCCCGCTAC |
| ***KASP primers*** | |
| ATM_652Wt | GAAGGTGACCAAGTTCATGCTTTTGGACTTTTTTTCCAAGGCTATTC |
| ATM_652Mut | GAAGGTCGGAGTCAACGGATTTTTGGACTTTTTTTCCAAGGCTATTT |
| ATM_652C | TACTGAGTCTAAAACATGGTCTTGC |
| ATM_995Wt | GAAGGTGACCAAGTTCATGCTCGGCAATATTACGAAATCCTGAAGAAT |
| ATM_995Mut | GAAGGTCGGAGTCAACGGATTCGGCAATATTACGAAATCCTGAAGAAC |
| ATM_995C | ACAACTTATATGATCTGCTAGTGAATG |
| ATM_1595Wt | GAAGGTGACCAAGTTCATGCTTGAACTTACCATGAAGGTCTGC |
| ATM_1595Mut | GAAGGTCGGAGTCAACGGATTCTGAACTTACCATGAAGGTCTGT |
| ATM_1595C | GGTAGTTTAGTTGAGGTTGACAGAGAA |
| ATM_5750Wt | GAAGGTGACCAAGTTCATGCTCACTCATTACATTACCTCTTTTGTCTTC |
| ATM_5750Mut | GAAGGTCGGAGTCAACGGATTCACTCATTACATTACCTCTTTTGTCTTT |
| ATM_5750C | CACTTTTTCCGATGCTGTTTGGATA |
| ATM_8560Wt | GAAGGTGACCAAGTTCATGCTGAGAAGCGATTGGCTTATACGC |
| ATM_856Mut | GAAGGTCGGAGTCAACGGATTGAGAAGCGATTGGCTTATACGT |
| ATM_8560C | CAAAAATAAAACCTGCCAAACAACAAA |
| CEP57_791Wt | GAAGGTGACCAAGTTCATGCTCTTCACACCTTTTCTGGTGGTTTTG |
| CEP57_791Mut | GAAGGTCGGAGTCAACGGATTCTTCACACCTTTTCTGGTGGTTTTC |
| CEP57_791C | TTGCAGACTGGTCTAGAAACAAATAGA |
| CHEK2_349Wt | GAAGGTGACCAAGTTCATGCTAGCAATATTCACAGCTTTTGTCCCT |
| CHEK2_349Mut | GAAGGTCGGAGTCAACGGATTAGCAATATTCACAGCTTTTGTCCCC |
| CHEK2_349C | CATGAAATTCAACAGCCCTCTGAT |
| CHEK2_593-1Wt | GAAGGTGACCAAGTTCATGCTAACCCATTTCTACTCTTTTCTTCCTTAG |
| CHEK2_593-1Mut | GAAGGTCGGAGTCAACGGATTAACCCATTTCTACTCTTTTCTTCCTTAT |
| CHEK2_593-1C | CCTTAGGATAAACTGACTGATCATCTACA |
| CHEK2_695Wt | GAAGGTGACCAAGTTCATGCTCCTCTAGTGGTGCCTGTGG |
| CHEK2_695Mut | GAAGGTCGGAGTCAACGGATTCCTCTAGTGGTGCCTGTGT |
| CHEK2_695C | TTGCTGATGATCTTTATGGCTACTTTC |
| BRIP1_847Wt | GAAGGTGACCAAGTTCATGCTCTATTCTTTCCAGCAGGGATCATACTT |
| BRIP1_847Mut | GAAGGTCGGAGTCAACGGATTCTATTCTTTCCAGCAGGGATCATACTC |
| BRIP1_847C | TTCTCATTTCTGTTGAAGTTACCGACT |
| FANCD2_2494+2Wt | GAAGGTGACCAAGTTCATGCTGTACTACCAGTATAGGACACTTCTCTTA |
| FANCD2_2494+2Mut | GAAGGTCGGAGTCAACGGATTGTACTACCAGTATAGGACACTTCTCTTG |
| FANCD2_2494+2C | TTGTAGAATTGCAAATAATCCTGGAAA |
| FANCI_206Wt | GAAGGTGACCAAGTTCATGCTTCCACCAACTGGATACAACAAGTGT |
| FANCI_206Mut | GAAGGTCGGAGTCAACGGATTTCCACCAACTGGATACAACAAGTGA |
| FANCI_206C | TAAGACTTGTTTCTGAACCCCCTGT |
| RAD51C_890Wt | GAAGGTGACCAAGTTCATGCTATAGAAATCAGGCCTTGCTTGTTC |
| RAD51C_890Mut | GAAGGTCGGAGTCAACGGATTATTGATAGAAATCAGGCCTTGCATTAGG |
| RAD51C_890C | AGAATCAAATGAAAGAGATAAGAAAAACTGTG |
| RECQL4_2636Wt | GAAGGTGACCAAGTTCATGCTCTGTGCCCAAGTACCCCCC |
| RECQL4_2636Mut | GAAGGTCGGAGTCAACGGATTCTGTGCCCAAGTACCCCCT |
| RECQL4_2636C | CTGTACGGTAAGCTGTATTGGGAGT |
| TP53_839Wt | GAAGGTGACCAAGTTCATGCTTGTTTGTGCCTGTCCTGGGAG |
| TP53_839Mut | GAAGGTCGGAGTCAACGGATTTGTTTGTGCCTGTCCTGGGAA |
| TP53_839C | CCTTTCTTGCGGAGATTCTCTTCCT |
| F- forward primer; R- reverse primer; Wt- primer specific for the wild-type allele; Mut- primer specific for the mutated allele; C- common primer. | |
